# Municipal Solid Waste Management and Adverse Health Outcomes: A Systematic Review

**DOI:** 10.3390/ijerph18084331

**Published:** 2021-04-19

**Authors:** Giovanni Vinti, Valerie Bauza, Thomas Clasen, Kate Medlicott, Terry Tudor, Christian Zurbrügg, Mentore Vaccari

**Affiliations:** 1 Department of Civil Environmental, Architectural Engineering and Mathematics, University of Brescia, 25123 Brescia, Italy; g.vinti001@unibs.it; 2 Gangarosa Department of Environmental Health, Rollins School of Public Health, Emory University, Atlanta, GA 30322, USA; valerie.bauza@emory.edu (V.B.); thomas.f.clasen@emory.edu (T.C.); 3 Department of Public Health, Environment and Social Determinants of Health, World Health Organization, 1211 Geneva, Switzerland; medlicottk@who.int; 4 SusConnect Ltd. Weedon Bec, Northamptonshire NN7 4PS, UK; terryl.tudor@gmail.com; 5 Department of Sanitation, Water and Solid Waste for Development (Sandec), Eawag—Swiss Federal Institute of Aquatic Science and Technology, Überlandstrasse 133, 8600 Dübendorf, Switzerland; Christian.Zurbruegg@eawag.ch

**Keywords:** MSW, public health, epidemiology, PRISMA guidelines

## Abstract

Municipal solid waste (MSW) can pose a threat to public health if it is not safely managed. Despite prior research, uncertainties remain and refurbished evidence is needed along with new approaches. We conducted a systematic review of recently published literature to update and expand the epidemiological evidence on the association between MSW management practices and resident populations’ health risks. Studies published from January 2005 to January 2020 were searched and reviewed following PRISMA guidelines. Eligible MSW treatment or disposal sites were defined as landfills, dumpsites, incinerators, waste open burning, transfer stations, recycling sites, composting plants, and anaerobic digesters. Occupational risks were not assessed. Health effects investigated included mortality, adverse birth and neonatal outcomes, cancer, respiratory conditions, gastroenteritis, vector-borne diseases, mental health conditions, and cardiovascular diseases. Studies reporting on human biomonitoring for exposure were eligible as well. Twenty-nine studies were identified that met the inclusion criteria of our protocol, assessing health effects only associated with proximity to landfills, incinerators, and dumpsites/open burning sites. There was some evidence of an increased risk of adverse birth and neonatal outcomes for residents near each type of MSW site. There was also some evidence of an increased risk of mortality, respiratory diseases, and negative mental health effects associated with residing near landfills. Additionally, there was some evidence of increased risk of mortality associated with residing near incinerators. However, in many cases, the evidence was inadequate to establish a strong relationship between a specific exposure and outcomes, and the studies rarely assessed new generation technologies. Evidence gaps remain, and recommendations for future research are discussed.

## 1. Introduction

Municipal solid waste (MSW) poses a threat to public health and the environment if it is not safely managed from separation, collection, transfer, treatment, and disposal or recycling and reuse. The World Health Organization (WHO) has highlighted the risks associated with the inadequate disposal of solid waste with respect to soil, water, and air pollution and the associated health effects for populations surrounding the involved areas [1].

Globally, MSW generation is expected to increase to 3.40 billion tonnes by 2050 [2]. In general, waste management practices tend to improve going from low-income to high-income countries [3,4]. As a consequence, the related health risks tend to be greater in low-income countries, where the most dangerous practices, such as open dumping and uncontrolled burning of solid waste, are still common [5]. Using published data, Vaccari et al. [6] compared characteristics of leachate from more than 100 landfills and dumpsites in Asia, Africa, and Latin America, and found statistically significant concentrations of pollutants in dumpsites.

Waste treatment and disposal includes recycling, composting, anaerobic digestion, incineration, landfilling, open dumping, and dumping in marine areas [2]. The impact of solid waste on health may vary depending on numerous factors such as the nature of waste management practices, characteristics, and habits of the exposed population, duration of exposure, prevention, and mitigation interventions (if any) [5,7,8]).

**Figure 1:**
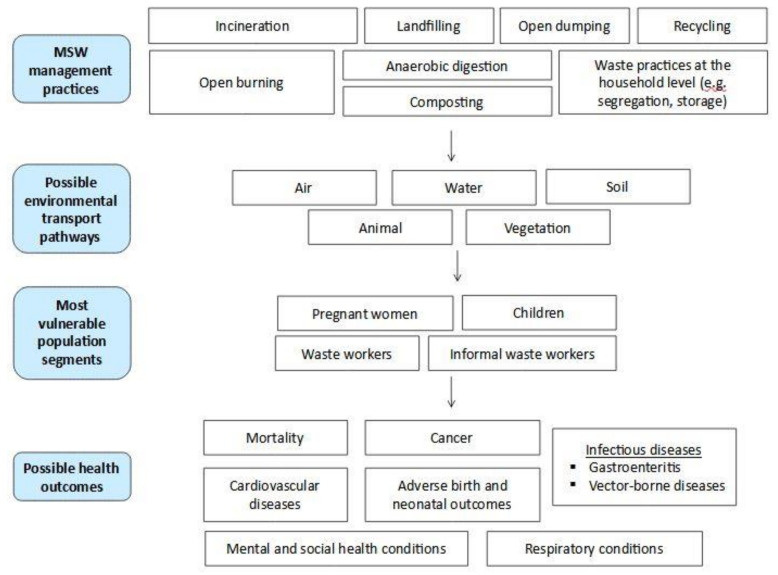
Schematic representation of the linkages between solid waste management practices and possible adverse health outcomes.

An investigation of the relationship between solid waste and human health begins with hazard identification and exposure assessment [1]. Figure 1 schematically represents the linkages between waste management practices, the respective hazards associated with these practices, the possible environmental pathways of transmission by which the most vulnerable or exposed population segments can absorb contaminants, and possible adverse health outcomes. Different waste management practices result in the release of different specific substances, including different environmental matrices that can be involved in transport and exposure. For example, air is the first environmental transport pathway for burning waste. By-products such as dioxins can be generated, and the ingestion of contaminated dairy products can represent an indirect source of exposure [9]. Other practices, such as waste disposal in landfills or dumpsites, can also affect groundwater through the leaking of leachate [10]; the consequent exposure would be represented by the ingestion of water contaminated with toxic or carcinogenic compounds [11].

Various reviews have explored the health effects related to solid waste management. Cointreau [12] published a detailed report on solid waste and health risks for population and workers, noting that the situation in low-income countries is usually worse. Cointreau’s work is probably the most exhaustive of the last 15 years. Porta et al. [13] examined epidemiological studies on health effects associated with management of solid waste, except for dumpsites and open burning areas. Mattiello et al. [14] analyzed the health effects focusing on people living nearby landfills and incinerators. Ashworth et al. [15] gathered data focusing on waste incineration and adverse birth outcomes. Ncube et al. [16] considered epidemiological studies related to municipal solid waste management, assembling the results based on the health risk (e.g., cancer, birth weight, congenital malformations, respiratory diseases), but this made difficult a comparison among MSW practices. None of these reviews analyzed studies published later than 2014. A further systematic review, recently published [17], focused on waste incinerators’ health impact, considering studies until 2017. In many cases, the authors suggested that MSW management practices can pose some adverse health effects for the population residing nearby, although the current evidence often lacked statistical power, highlighting the need for further investigations. At the same time, with a moderate level of confidence, some authors derived effects from old landfills and incinerators, such as an increased risk of congenital malformation within 2 km for landfills and cancer within 3 km for incinerators [13]; other authors [14] found an increased risk of congenital anomalies mainly nearby special waste landfills, and regarding incinerators some authors found some limited risks of cancer and birth defects, highlighting changes in technology are producing more reassuring results [14]. Still, the previous reviews rarely analyzed the changing operational standards associated with the evolving legislation. Although their approach can represent a prudent strategy, it limited the interpretation of some data. Only Mattiello et al. [14] conducted this type of analysis.

Focusing on composting facilities, two systematic reviews analyzed health outcomes, but only considered bioaerosols exposure [18,19]. In both studies, the authors concluded that there is insufficient evidence to provide a quantitative comment on the risk to nearby residents, although there is sufficient evidence to support a precautionary approach, and further research is needed.

In most of the reviews mentioned above, vector-borne diseases (such as malaria) were not included. Only Ncube et al. [16] cited one study about malaria [20] and Cointreau [12] mentioned a couple of old studies related to vector-borne diseases. Although one recent review [21] focused on the link between solid waste and vector-borne diseases, the methodology and results did not follow a systematic procedure, and appeared excessively approximate.

Additionally, the PRISMA methodology, characterizing a recently recommended systematic review approach [22,23], was rarely implemented. Only in the works of Pearson et al. [18], [19] and Tait et al. [17] was it applied, i.e., in studies that only involved a specific solid waste management practice.

Therefore, despite such prior reviews, uncertainties remain. In many cases, how future research should be developed was not addressed enough. Additionally, the influence of national legislation, characterizing operational standards and technological level, was rarely investigated. Furthermore, WHO [1] noted that the health effects of waste management and disposal activities are only partly understood. In some cases, it is challenging to apply estimates and evidence from studies related to high levels of emissions from the past to new-generation incineration plants. It has to be highlighted that solid waste legislation influences the technological level and emission limits associated with solid waste management plants, such as landfills and incinerators. Indeed, in many European countries, modern technology has been reducing noxious emissions, and measurable health impacts have, in many cases, become smaller. For example, even the review of Tait et al. [17], in which the authors focused on incinerators’ publications until 2017, should be renewed, based on more recent and robust studies (e.g., [24,25]). At the same time, it has to be considered that the so-called emerging contaminants (ECs) are not commonly monitored in the environment, but they have the potential to enter the environment and cause known or suspected adverse health effects [26]. In addition, many new chemicals are constantly approved for commercial use; for example, over 40,000 chemicals are actively being manufactured, processed, and imported in the United States, but the health effects of few of them have been monitored in the population [27,28]. Such substances can easily reach the solid waste phase, leading to underestimated adverse health outcomes. Besides, countries with weak environmental legislations can be affected by additional risks. For instance, some persistent organic pollutants (POPs) are still in production and use in countries that have not ratified the Stockholm Convention, such as in Southern Asia [29]. Consequently, updated evidence is needed for the policy debate.

Thus, we have undertaken the present systematic review in order to update and expand on previous reviews, based on the PRISMA statement [23]. Specifically, the objective was to assess and summarize the evidence on the association between municipal solid waste (MSW) management practices and health risks to populations residing nearby. Data were gathered and analyzed in a different way compared with the studies aforementioned. After summarizing the results, the findings are discussed in detail in the Discussion section, considering the influence of national legislation and the technological level in the case of landfills and incinerators. It represents the main novelty of the topic. Furthermore, the update of the recent scientific literature related to MSW and health outcomes using the PRISMA statement was provided, also taking into consideration that some categories, such as dumpsites and vector-borne diseases, were not adequately analyzed in previous reviews. Such a comprehensive approach represented an added value to the manuscript. Finally, we also discussed how further research should be conducted.

## 2. Methods

The methods used in this review were developed based on the Preferred Reporting Items for Systematic Reviews and Meta-Analyses (PRISMA) statement [22,23]. The PRISMA is a procedure that originated in 2009, consisting of a 27-item checklist and a PRISMA flow diagram [23] that helps authors develop the systematic review in a well-structured way to address recent advances in the science of systematic reviews. The complete procedure is available in the protocol registered on PROSPERO [30], an international database of prospectively registered systematic reviews.

### 2.1. Definitions

Some of the technical terms used in this review are defined below.

- Municipal solid waste (MSW): any material from residential, commercial, and institutional activities which is discarded. It is important to note that industrial, medical, hazardous, electronic, and construction and demolition wastes belong to other categories [2].
- Engineered landfill: site characterized by the registration and placement/compaction of waste. Such landfills typically use daily cover material, surface and ground water monitoring, infrastructure, and a waterproof liner at the bottom [6].
- Sanitary landfill: site characterized by the registration and placement/compaction of waste. Best practices include a waterproof liner at the bottom, leachate and gas collection systems, daily cover, a final top cover and closure, infrastructure as well as a post-closure plan [6].
- Dumpsites: open and unregulated areas or holes in the ground with no environmental protection and disposal controls [6]. Due to lack of controls, dumpsites may receive different waste streams including MSW, sewage sludge, hazardous waste, electronic waste, healthcare waste [31].
- Transfer stations: facilities in which waste is transferred from smaller vehicles used for waste collection into bigger vehicles for hauling to a disposal or treatment site [32].
- Incinerators: a specialized engineered system where waste is burned. Through combustion waste is converted into ash, flue gas, and heat. The flue gases are treated to reduce impact of air pollution on environment and health. Energy from an incinerator can be recovered [32].
- Open burning of waste: burning of solid waste in open areas without air pollution controls [32].

Dumpsites and open burning were categorised together since burning waste in dumpsites is a common practice, especially in low- and middle-income countries [5,12], making it impossible to split it into two separate categories. As the definition of dumpsites suggests, it was not always possible to assure a clear distinction between MSW and other categories of waste. As a consequence, dumpsites were excluded in cases where the sites did not receive MSW but only other categories of solid waste. Furthermore, in many cases it was not possible to find a clear distinction between sanitary and engineered landfills among the publications, as a consequence the two categories were combined. However, as will be discussed later, such definitions of landfills and incinerators need to be contextualised. Indeed, the fast-evolving technologies and more restrictive legislation [1] can influence the emission limits and the related health outcomes.

### 2.2. Study Eligibility

As detailed more fully in the review protocol, studies were eligible for inclusion in the review if they met specified criteria for population, exposure, and health effects. The eligible population and exposures were persons, both children and adults, living, studying, or spending time near MSW treatment or disposal sites, such as landfills, dumpsites, incinerators, areas in which open burning of waste is conducted, transfer stations, recycling sites, composting plants, and anaerobic digesters. Eligible comparators were residents who were not exposed, residents with a lower level of exposure and residents located at different distances from MSW treatment or disposal sites. Occupational risks and therefore waste workers (regular or informal) were not assessed, because they were related to a further category, subjected to different exposures also in terms of time. Health effects included mortality, adverse birth and neonatal outcomes, respiratory conditions, cancer, gastroenteritis, vector-borne diseases, mental and social health conditions, and cardiovascular diseases. Studies reporting on human biomonitoring for exposure were also eligible. The inclusion of transfer stations and vector-borne diseases [33] as an outcome was a modification from the pre-specified protocol submitted to PROSPERO. However, no changes were made to the search strategy as a result of this addition.

Randomized controlled trials (RCTs) and the following non-randomized controlled studies (NRS) were included: quasi-RCTs, non-RCTs, controlled before-and-after studies, interrupted-time-series studies, historically controlled studies, case-control studies, cohort studies, and cross-sectional studies that include a comparison group. Studies were excluded if they reported qualitative data only.

To be eligible for inclusion, studies had to be peer reviewed and published in English.

### 2.3. Search Strategy; Screening and Data Extraction; Narrative Review

The search for eligible studies was conducted using relevant search engines (i.e., Scopus, ScienceDirect, Google Scholar) with a combination of keywords based on possible MSW exposure and health effects. Further details regarding the electronic search strategy, including the keywords and string, are available in the protocol. Studies published from January 2005 to January 2020 were examined.

Following an initial screening of paper titles and abstracts, the full paper was examined for eligibility by a single reviewer. Thereafter, data were extracted from eligible studies and compiled solely from the paper.

Due to substantial differences between the studies included in terms of settings, populations, study designs, contexts, MSW management practices, exposure assessment, case definitions, outcome definitions and outcome assessment, it was determined that a pooled analysis using meta-analysis or meta-regression was not appropriate. Accordingly, this review adopted a narrative approach.

### 2.4. Risk of Bias; Quantity and Strength of Evidence

One reviewer assessed the risk of bias associated with experimental studies, based on the Liverpool Quality Assessment Tool (LQAT), an adaptation of the Newcastle-Ottawa Scale [34]. Observational studies were automatically scored as having a very serious risk of bias due to the many potential sources of bias inherent in the study design.

Finally, the strength of evidence was summarized to develop the different health outcomes as a function of the categories of exposure analyzed (e.g., landfills, dumpsites). The following values were given: (0) no studies; (−) studies, but no evidence of increased risk; (+) studies, providing some evidence of increased risk; (++) studies, with stronger evidence of increased risk. The findings are discussed in detail in the discussion section taking also into consideration the technological level of the units in the case of landfills and incinerators.

## 3. Results

### 3.1. Study Selection

**Figure 2:**
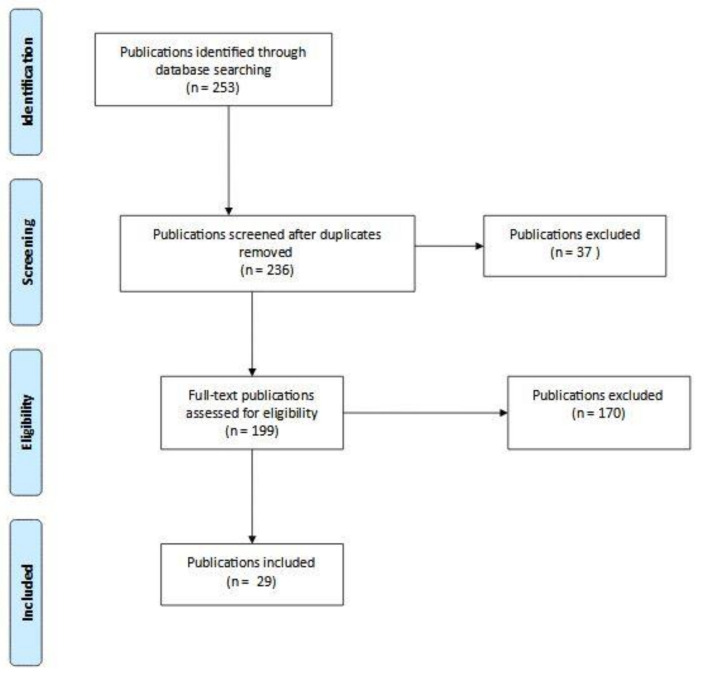
PRISMA flow diagram summarizing the study selection.

A total of 253 studies, including 33 reviews and reports, were initially identified. After adjusting for duplicates, 236 remained. Of these, 37 studies were discarded after reviewing the abstracts (if any) because it appeared these papers clearly did not meet the criteria. The full text of the remaining 199 publications was examined in more detail. A total of 170 studies did not meet the inclusion criteria previously described. Twenty-nine studies met the inclusion criteria and are included in this review. The PRISMA flow chart describing the process for determining study eligibly appears in Figure 2 below. All studies screened are available in Supplementary Materials.

### 3.2. MSW Transfer and Treatment Sites

Although the review sought to summarize studies investigating health effects associated with MSW transfer and treatment sites, we did not identify any eligible studies. Specifically, no studies were found that met the review’s inclusion criteria for health effects associated with proximity to transfer stations, recycling centers, composting plants, and anaerobic digesters.

### 3.3. MSW Disposal Sites

Table 1, Table 2, Table 3, Table 4, Table 5 and Table 6 summarize the results. In particular, in terms of the methodology used in each paper, the results concerning MSW disposal sites are summarized in Table 1 (landfills), Table 3 (incinerators) and Table 5 (dumpsites and open burning). The studies are listed in alphabetical order by author. In terms of health outcomes, the results are summarized in Table 2 (landfills), Table 4 (incinerators), and Table 6 (dumpsites and open burning). In Table 2, Table 4 and Table 6, the results are gathered based on the eight categories of health outcomes previously mentioned (i.e., mortality, adverse birth and neonatal outcomes, respiratory conditions, gastroenteritis, vector-borne diseases, mental and social health conditions, cardiovascular diseases, human biomonitoring). Consequently—in Table 2, Table 4 and Table 6—the same research can be cited multiple times if different outcomes were assessed within the same study. Additionally, when an adverse health effect resulted in *p* < 0.05, it was bolded within the table. However, the publications rarely mentioned technological elements and emission limits characterizing landfills and incinerators in the case study. Therefore, we carried out an additional investigation to address this aspect.

#### 3.3.1. Landfills

We identified nine studies relating to landfills (Table 1). These were mainly conducted in Europe (5) and North America (2). Only one was from Asia (China) and one from Africa (South Africa). Five papers were retrospective cohort studies and four were cross-sectional studies.

The overall evidence of health risks associated with residing near a landfill is mixed (Table 2). Considering results with a significance of *p* < 0.05, there is some evidence increased risk of mortality for lung cancer [35], births with congenital anomalies [36], and negative respiratory conditions in people aged ≤14 years, considering both all respiratory diseases and only acute respiratory infections [35], association between increase of PM_2.5_ concentration and reduction of forced vital capacity in children aged 6–12 years [37], mucosal irritation and upper respiratory symptoms [38], and other mild symptoms [39,40]. There was also some evidence of worsening mental and social health conditions, such as alteration of daily activities or negative mood states [38]. Other studies, however, found no evidence of mortality or adverse health effects. Indeed, Mataloni et al. [35] did not find evidence of increased mortality for other specific cancers (i.e., colorectal, kidney, liver, pancreas, larynx, bladder, stomach, brain, and lymphatic tissue) as well as for cardiovascular, digestive, ischemic heart, respiratory, and urinary system diseases. For congenital anomalies, no evidence of increased cases was found by Elliott et al. [41]. Jarup et al. [42] found no evidence of increased risk of birth with Down’s Syndrome. No evidence of increased specific cardiovascular diseases (cardiac, ischemic, and cerebrovascular) was found by Mataloni et al. [35]. Neither evidence of increased risk of asthma [35,39] nor gastrointestinal symptoms [38] was found.

#### 3.3.2. Incinerators

Table 2 summarizes the evidence related to incinerators. A total of 13 studies were identified, 10 of which were conducted in Europe and three in Asia. Seven papers were retrospective cohort studies, one was a prospective cohort study, three were case-control studies and two were cross-sectional studies.

Considering results with a significance of *p* < 0.05, like landfills, the evidence of increased health risks from residing near an incinerator is mixed. A study reported increased risk of mortality in women for various health outcomes, including cancer [44]. There is also evidence of adverse birth and neonatal outcomes—i.e., preterm births [45], congenital heart defects, genital system defects and hypospadias [25], urinary tract birth defects [46]. Furthermore, human biomonitoring studies suggest higher levels of dioxins found in residents near incinerators [9,47]. Other studies, however, found no evidence of adverse health effects. In particular, Viel et al. [48] found no evidence of increased invasive breast cancer in women aged 20–59 years, even founding a significant reduction in invasive breast cancer in women aged 60 years and over. Ranzi et al. [44] found no evidence of increased cancer diseases both in men and women. Several studies reported no evidence of many adverse birth outcomes [24,25,45,46,49,50,51]. Ranzi et al. [44] found neither evidence of increased risk of cardiovascular diseases nor respiratory issues. There was also no evidence of increased mortality in men for various health outcomes, including cancer.

#### 3.3.3. Dumpsites and Open Burning

Table 3 summarizes the effects of residing near dumpsites and open burning. This includes a total of seven studies, one of which was carried on in Latin America, two in North America and four in Africa. Three were retrospective cohort studies, and four were cross-sectional studies.

Once again, the evidence of adverse health effects from the exposure is mixed. Considering results with a significance of *p* < 0.05, there is some evidence suggesting that residing near dumpsites is associated with increased risk of adverse birth or neonatal outcomes in terms of low birth weight [58]. However, most studies found no evidence of adverse health effects, including mortality [59], and congenital malformations [60]. In terms of gastroenteritis, all studies were from Africa and cross-sectional [20,61,62,63], but the results were mixed and not statistically significant. Malaria was the only vector-borne disease that studies were identified for. The same four studies that reported on gastroenteritis also reported on malaria, and the evidence suggested that there may be an increased risk of malaria for nearby residents, although none of the results were statistically significant.

### 3.4. Study Quality

All studies that met the established inclusion criteria for this review were observational studies, and thus were automatically scored as having a very serious risk of bias due to the many potential sources of inherent bias with these study designs. In particular, many included studies suffered from deficiencies such as lack of control for potential confounders, small sample size, unclear case definitions, reliance on self-reported data, and/or the inclusion of several different health outcomes which could increase the type I error rate.

### 3.5. Summary of Results

Table 7 summarizes the quantity and strength of the evidence related to MSW sites and health outcomes by type of MSW exposure and outcome. In general, there is a paucity of evidence, with no studies for certain exposures and outcomes. This is particularly true in the case of mental health and social health conditions and in biomonitoring, and for most health outcomes associated with dumpsites and open burning. Only mortality and adverse birth outcomes have at least one study for each type of exposure.

In addition to the dearth of evidence, the results are mixed. There was evidence to suggest an increased risk of adverse birth and neonatal outcomes for all types of MSW sites, whereas for other outcomes there was either a lack of evidence for one or more MSW site type or varied evidence of health effects for different kinds of MSW sites. There was also some evidence of health outcomes for landfills and incinerators compared to dumpsites or open burning sites. However, legislation that could characterize landfills and incinerators in each country should be taken into account. This aspect is addressed in the Discussion section below.

## 4. Discussion

We conducted a systematic review of literature published within the past 15 years (January 2005 to January 2020) to assess and summarize the epidemiological evidence on the association between MSW treatment or disposal sites and health risks to resident populations. The 29 studies that met the inclusion criteria investigated the health effects associated with living nearby landfills (9 studies), incinerators (13 studies), and dumpsites or open burning sites (7 studies). Health outcomes included a large range of conditions, including mortality, cancer, adverse birth and neonatal conditions, cardiovascular diseases, respiratory conditions, gastroenteritis, vector-borne diseases, and mental health conditions. Three studies reported on biomarkers of disease rather than actual health conditions.

Overall, the results were mixed or limited. The most consistent evidence was on the adverse birth and neonatal outcomes, with studies identifying increased risks associated with living near all three types of MSW disposal sites. There was some evidence of increased risk of mortality associated with living near landfills or incinerators. We found no evidence suggesting an increased risk of cancer, cardiovascular diseases, gastroenteritis, or vector-borne diseases. There were no studies on these outcomes in respect of landfills or dumpsites and cancer, dumpsites/burning and cardiovascular diseases, or incinerators and gastroenteritis, and landfills or incinerators and vector-borne diseases. Mental health conditions were investigated only in the case of landfills, where there was evidence of adverse effects. Similarly, human biomonitoring was explored only in the case of incinerators where there was evidence of an increased level of PCDD/F in children’s blood and mother’s breast milk in studies in China [9,47] but not in Spain [53]. As outlined, the publications rarely mentioned technological elements and emission limits regarding solid waste management for the case studies. Therefore, we carried out additional investigations to fill this gap.

With respect to proximity to landfills, there was evidence of an increased risk of congenital anomalies in a retrospective cohort study by Palmer et al. [36]; while in another cohort study Elliot et al. [41] did not find evidence of increased risk. However, Palmer et al. [36] and Elliot et al. [41] studied landfills that were operational between the early 1980s and the late 1990s in the UK. Landfills in the UK were regulated by the Control of Pollution Act [64], replaced by the Waste Management Licensing Regulations in 1994 [65], and, the UK only fulfilled the European Landfill Directive [66] to improve standards and reduce adverse effects on the environment in 2002. As a consequence, the two studies were related to the impact of old landfills, i.e., from the previous generation used in the UK. There appears to also be an increased risk in mortality for lung cancer and respiratory diseases, as well as increased morbidity related to respiratory diseases, mainly among youths and children [35,37,38]. In particular, Mataloni et al. [35] considered the association to landfill H_2_S exposure (used as a tracer in the air). When they repeated the analysis using the distance from landfill instead of H_2_S concentration, there were no significant associations between mortality outcomes and living 0–2 km from a landfill compared to 3–5 km. Models that consider the pathways of contaminants instead of only focusing on the distance are likely more accurate. However, Mataloni et al. [35] considered the health effects of landfills in Italy between 1996 and 2008, and the European Landfill Directive [66] was implemented in 2003 [67] in Italy, and by 2009 the landfills that were already operational had to be adapted to the new legislation. All landfills included in Mataloni et al. [35] were activated before the new Italian legislation. Consequently, it can be assumed that the findings refer to the effect of the old generation landfills in the country. Furthermore, the study of Gumede and Savage [37] was carried out in South Africa, in which the operational standards related to landfills are less restrictive than the most recent European directives [68]. In addition, Heaney et al. [37] found an increased risk of alteration of daily activities and negative mood states, but the cross-sectional study included only 23 participants. However, the research of Heaney et al. [38] was carried out in North Carolina (USA) in 2009, but the Federal Regulation concerning MSW landfills was revised in 2011, addressing some major aspects including operating practices and composite liners requirements [69]. Therefore, even in this case, the adverse health outcomes related to new generation landfills in the USA could be lower. In the studies included in this systematic review, there was no other evidence of increased risks related to other kind of diseases. In addition, it must be noted that none of the studies on landfills explicitly focused on potential leachate pollution and related human health risks. Indeed, even modern landfills with good quality geomembranes can sometimes leak leachate due to thermal expansion of the material, folds generated during installation or initial defect density, causing potential risk for water bodies and its consumers; as a consequence, the risks related to landfills are not only due to air emissions [70].

Likewise, there is mixed and limited evidence on the health effects associated with living near incinerators. It is also important to consider the type of incinerators and emissions control technologies being implemented when assessing health effects. MSW incinerators operating in Europe before the Waste Incineration Directive [71] can be considered from the old generation of incinerators. After the implementation of the directive, that existing plants needed to comply with by the end of December 2005, the corresponding incinerators can be assumed to be from the new generation. Further improvements were made in 2018 when the new Best Available Techniques (BATs) for waste treatment was adopted by the European Commission [72], and the MSW incinerators that were already operational have four years to comply with the new standards. Thus, the last category can be assumed as the newest generation, for which no epidemiological studies exist. Regarding the research included in Table 4, two retrospective cohort studies [24,45] assessing European incinerators between 2003 and 2010 obtained different results for preterm births. Compared to Ghosh et al. [24], Candela et al. [45] used a smaller buffer zone around each incinerator, namely 4 km instead of 10 km. According to Ghosh et al. [24] this difference in approach may have led to fewer outcomes with a lower estimated exposure included. Additionally, in Candela et al. [45], which was carried out in Italy, the estimated annual average exposure to PM_10_ from incinerators in the study areas was 0.96 ng/m^3^ in 2003, decreasing to 0.26 ng/m^3^ in 2010 because of the improvements of the incineration plant during the study period. However, the annual average exposure to PM_10_ estimated in Ghosh et al. [24] was in the same order of magnitude. In terms of birth with congenital anomalies of the genital system, Parkes et al. [25] found an association with distance from incinerators but not PM_10_. Ghosh et al. [24] and Parkes et al. [25] assessed an intermediate period between old and new generation plants; indeed, for the existing plants the new directive became operational in the end of December 2005. Therefore, although the epidemiological studies mentioned above are among the widest and most recent, their findings can be assumed to be a transition period, between old and new generation plants. Updated research is necessary, only focusing on emissions from new and newest generation plants. In a retrospective cohort study involving residents in Forly (Italy), Ranzi et al. [44] found a general higher rate of mortality in women and also a higher rate of mortality considering all types of cancer in women. However, the authors analyzed a cohort of people until 2003. As a consequence, the results are only related to old generation plants. Furthermore, Cordier et al. [46] found an increased risk of urinary track birth defects (UTBD) in infants exposed to MSW incineration dioxins (both atmospheric and deposits). In addition, the findings of Cordier et al. [46] suggested that consumption of local food modified the risk, increasing it in exposed areas. However, the authors analyzed the outputs between 2001 and 2004; therefore, the incinerators belonged to the old generation sites [73]. Noteworthy, Parkes et al. [25] found no evidence of increased risk of UTBD, and their study analyzed more recent incinerators. Regarding biomonitoring studies, Xu et al. [9,47] found higher levels of dioxins in residents near incinerators in China. In contrast, the values from a study conducted in Spain [53] were uncertain, varying over the years and often being greater in unexposed groups. However, it is important to highlight in the studies of Xu et al. [9,47] that the samples were collected in China in 2013, i.e., before the approval of more restrictive legislation for MSW incinerators emissions in 2014 [74]. The new Chinese legislation has standards comparable to those of the European Union [74]. Consequently, updated studies are necessary.

As highlighted in the studies discussed above, the definitions of landfills and incinerators need to be contextualized based on the evolving technologies and national/international legislation [1]. For example, European incinerators’ current emission limits are more restrictive than a couple of decades ago. Therefore, many health outcomes related to such new generation plants appear to be lower than in the past. However, the results from such old generation plants can continue to be suitable in areas where less restrictive limits continue to be applied, such as in some developing countries [75].

Many results are also consistent with the systematic review of Ncube et al. [16], in which the authors found landfills and incinerators presented adverse health endpoints even if epidemiological evidence in reviewed articles were often inadequate. However, as discussed above, although the operational standards have changed over time, they were not considered by Ncube et al. [16].

As many dumpsites also practice open burning, it was not possible to assess the effects of these separately. An increased risk of adverse birth outcomes was found for low birth weight and intrauterine growth retardation. However, the main related study [58] did not expressly specify if the dumpsites were all for MSW. The lack of studies on dumpsites and open burning is especially noteworthy given the widespread prevalence of these methods for disposing of MSW [5].

In addition, four studies assessed the association between vector-borne diseases and dumpsites [20,61,62,63]. Although these were cross-sectional studies with small sample sizes, making the evidence too weak to link to an increased risk, they analyzed important health outcomes rarely taken into account. Besides, an increased risk of malaria in people residing closer to dumpsites was noted by some authors [20,62,63], offering some suggestive evidence of this adverse health effect. Still, more robust studies are needed.

Overall, many of the studies that were identified and included in this review were of low quality, therefore the potential for causal inference from the studies is limited. While randomized controlled trials of these conditions are probably not possible, there may be opportunities for future studies to use natural experiments or time series analyses. All of the included studies followed observational study designs and presented significant potential for bias and confounding. For example, important measures of exposure such as length of time, activity, technological characteristics, and distance to the hazard, were not always controlled. Case definitions were not always clear, and the methods for case ascertainment in some cases was reported rather than clinically confirmed. In addition, given the range of types of studies and the exposures and outcomes measured, the use of a narrative, as opposed for example, to a meta-analysis or meta-regression was effective in searching, screening, and extracting the necessary data for the review.

This review focused on health effects associated with residing near MSW sites and our findings are limited to only nearby resident populations. A limitation of this work is that it does not consider the health of the larger community in relation to solid waste management or the differential health effects associated with varying levels of MSW management. For example, even if there are some negative health risks for nearby residents of MSW sites, appropriate solid waste management could overall be helpful for the health of populations at large. Living near unmanaged solid waste could also lead to greater negative health impacts than living near a managed solid waste site and this review did not perform a comparative analysis for different types of solid waste management situations (such as no waste management, poorly managed MSW sites, well management MSW sites, and reduced waste generation).

In future, in addition to epidemiological studies, consideration should be given to conducting biomonitoring research. Indeed, focusing on the burning of solid waste (both in incinerators and through uncontrolled open burning) most general population exposure to dioxin (PCDD/F) is through ingestion of contaminated foods of animal origin [55], with approximately 80–90% of the total exposure via fats in fish, meat, and dairy products [76]. Generally, levels of dioxins in air are very low, except close to sources such as inefficient incinerators or open burning. Releases into the air ends up contaminating soil and aquatic sediments and can lead to bioaccumulation and bioconcentration through food chains [55]. Furthermore, dioxins decompose very slowly in the environment, remaining there for very long periods [76]. Thus, the biomonitoring of the presence of dioxins as well as other persistent pollutants in farm animals and their derivatives nearby incinerators would be useful. Some works have already been carried out and can be taken as references for future research. For example, Cordier et al. [46] analyzed the association between local food consumption, dioxin deposits generated by MSW incinerators and risk of urinary tract birth defects. More recently, Xu et al. [9] studied the concentration of dioxins on eggs close to an MSW incinerator in China.

In addition, the biomonitoring studies should be extended to other waste practices. The work of Scaramozzino et al. [77] can be considered as well. The authors conducted the first proposal for a standardized protocol for farm animal biomonitoring that can be useful for both environmental and human risk assessments.

Furthermore, technical aspects influenced by national legislation should be investigated further. This would allow for easier comparisons between evolving technologies for which environmental and health impacts tend to decrease.

## 5. Conclusions

In conducting this systematic review, 29 studies were identified that met the inclusion criteria of our protocol, assessing health effects only associated with proximity to landfills, incinerators, and dumpsites/open burning sites. Compared to most previous reviews, national legislation’s influence—characterizing operational standards and technological level—was investigated. There was some evidence of an increased risk of adverse birth and neonatal outcomes for residents near landfills, incinerators, and dumpsites/open burning sites. There was also some evidence of an increased risk of mortality, respiratory diseases, and negative mental health effects associated with residing near landfills. Additionally, there was some evidence of increased risk of mortality associated with living near incinerators. However, in many cases, the evidence was inadequate to establish a strong relationship between a specific exposure and outcomes. Additionally, most landfills and incinerators investigated referred to the old generation of technologies, although studies on new generations’ plants are starting to be published. Therefore, future research should focus on new generation landfills and incinerators, to have a more specific analysis of these upgraded MSW practices. Additionally, the health effects related to the open burning of waste need further investigation, and the association between dumpsites in developing countries and vector-borne diseases require more robust epidemiological studies.

However, none of the 29 studies that we identified investigated the health effects associated with MSW transfer and treatment, such as transfer stations, recycling centers, composting plants, and anaerobic digesters. This appears to be a major gap in the literature since transfer and treatment facilities are widespread and could pose health risks including exposure to toxins, particulate or infectious agents via direct contact, and aerosolization or other pathways. Since these health risks are potentially different from those associated with MSW disposal sites, future research must address this gap to assess relative risks associated with various management and disposal options.

## Figures and Tables

**Table 1:** Landfills—methodology characterizing each research.

Study Location	Study Design	Study Participants	Study Period	Exposure Source	Outcomes Investigated	Ref.
England (UK)	Cohort study (retrospective)	10,064,382 live births, 52,532 stillbirths and 12,373 terminations	Births between 1983 and 1998	8804 landfills, including 607 which handled special (hazardous) waste	The risk of congenital anomalies in relation to an index of geographic density of landfill sites (within 2 km from landfills)	[41]
South Africa	Cross-sectional study	23 children aged 6–12 years residing within 2 km from the landfill site for at least 5 years	Study conducted between November 2013 and January 2014	The Bisasar Road MSW landfill	Assessment of PM_2.5_ concentration in indoor environments of the subjects involved in the study and its association with lung function patterns	[37]
North Carolina (USA)	Cross-sectional study	23 participants among people living within 0.75 miles to a landfill	Between January and November 2009	A MSW landfill	Relationships between H_2_S, odour, and health outcomes in a community living close to a landfill	[38]
England and Wales (UK)	Cohort study (retrospective)	4,584,541 births in England and Wales	Births between 1989 and 1998	6289 landfill sites processing special (hazardous), non-special and unknown waste	The risk of giving birth to a child with Down syndrome associated with residence near landfill sites (within 2 km)	[42]
Denmark	Cohort study (retrospective)	2477 live births with congenital anomalies in Denmark in three different zones of distance from landfills (0–2 km; 2–4 km; 4–6 km)	Births between 1997 and 2001	48 landfills	Risk of congenital anomalies combined and congenital anomalies of the cardiovascular and nervous systems with maternal residence in function of distance from landfills	[43]
Missouri (USA)	Cross-sectional study	Health survey through 170 households within a 3.2-km radius from a landfill and 173 households more distant (comparison group) from the landfill	Conducted from February to March 2016	The Bridgeton Landfill in St. Louis County, in which MSW is disposed of	Respiratory symptoms and diseases were assessed, though household interviews	[39]
Italy	Cohort study (retrospective)	242,409 people living within 5 km from landfills	Residents between 1996 and 2008, followed for mortality and hospitalizations until 2012	9 MSW landfills operating in the Lazio region, in which the exposure to landfills was assessed using H_2_S as a tracer in air (calculated with a model)	The association between landfill H_2_S exposure and mortality (both natural and cause-specific) and hospital admissions for cardiorespiratory diseases was evaluated	[35]
Wales (UK)	Cohort study (retrospective)	542,682 births in Wales between 1983 and 1997. 97,292 births in Wales between 1998 and 2000	See previous column	24 landfill sites for commercial, industrial, and household waste	The increased risk of births with at least one congenital malformation in population living within 2 km from landfill sites, comparing it with population living at least 4 km away	[36]
China	Cross-sectional study	951 children from primary school studying and residing near a landfill. 4 schools within 5 km of the landfill (exposed area). 1 school (non-exposed area) more distant (5.8 km away)	Not specified	A MSW landfill	Association between air pollutants and respiratory health in exposed area, considering lysozyme and secretory immunoglobulin A (which are typically considered as the first line of defence from air pollutants and higher levels show good related health conditions)	[40]

**Table 2:** Health outcomes associated with landfills.

Study Location	Study Design	Main Findings (e.g., Estimated Risk, CI, *p*-Value)	Ref.
***Mortality***			
Italy	Cohort study (retrospective)	Associations between H_2_S (>75° quartile) and cause-specific mortality (hazard ratio (HR) and 95% Confidence Interval): - natural cases: 0.98 (0.91, 1.05) - all cancers: 1.03 (0.91, 1.16) - specific cancers:stomach: 0.88 (0.54, 1.42) - colorectal: 0.91 (0.64, 1.28) - liver: 0.76 (0.48, 1.2) - pancreas: 0.73 (0.41, 1.32) - larynx: 0.26 (0.07, 0.95) - **lung: 1.34 (1.06, 1.71), *p* < 0.05 ^a^** - bladder: 0.94 (0.5, 1.80) - kidney: 0.86 (0.41, 1.83) - brain: 1.76 (0.81, 3.81) - lymphatic and hematopoietic - tissue: 1.12 (0.74, 1.17) - tissue: 1.12 (0.74, 1.17) - cardiovascular diseases: 0.91 (0.81, 1.02) - ischemic heart diseases 0.78 (0.64, 0.95) - respiratory diseases: 1.30 (0.99, 1.70) - digestive diseases: 0.97 (0.69, 1.35) - urinary system diseases: 1.42 (0.84, 2.40)	[35]
***Adverse birth and neonatal outcomes***			
England (UK)	Cohort study (retrospective)	Rates of congenital anomalies in the category with the highest exposure index (the fourth), for non-special or unknown waste sites (adjusted odds ratio (OR) and 95% Credible Interval): - all congenital anomalies (hypospadias and epispadias, cardiovascular defects, neural tube defects, abdominal wall defects): 1.02 (0.98, 1.07) - hypospadias and epispadias: 0.97 (0.89, 1.06) - neural tube defects: 1.04 (0.93, 1.18) - cardiovascular defects: 0.94 (0.82, 1.07) - abdominal wall defects: 1.11 (0.94, 1.32)	[41]
Denmark	Cohort study (retrospective)	Risk rate ^b^, comparing the closest zones with the others. When RR < 1.000 the risk is lower, compared to the closest zone: - combined congenital anomalies: 1.000 (closest zone), 0.991 (middle zone), 1.013 (farthest zone) - congenital anomalies in the cardiovascular system: 1.000 (closest zone), 0.926 (middle zone), 0.854 (farthest zone)	[43]
England and Wales (UK)	Cohort study (retrospective)	Relative risk (RR) ^c^ (95% Credible Interval) of Down’s syndrome near landfill sites: - considering both operating and closed sites (non-special waste): 1.000 (0.909, 1.095) - considering only operating sites (non-special waste): 1.011 (0.901, 1.126)	[42]
Wales (UK)	Cohort study (retrospective)	Ratio between risk of congenital anomalies (in live births) after and before opening of sites (95% Confidence Interval): **1.39 (1.21, 1.72), *p* < 0.05 ^a^**	[36]
***Cardiovascular diseases***			
Italy	Cohort study (retrospective)	Associations between H_2_S (>75° quartile) and cardiorespiratory morbidity (HR and 95% Confidence Interval): - (all) cardiovascular diseases: 1.02 (0.97, 1.07) - cardiac disease: 1.04 (0.97, 1.11) - ischemic heart diseases: 0.99 (0.88, 1.10) - cerebrovascular diseases: 0.98 (0.88, 1.10)	[35]
***Respiratory conditions***			
Italy	Cohort study (retrospective)	Associations between H_2_S (>75° quartile) and cardiorespiratory morbidity (HR and 95% Confidence Interval): - (all) respiratory diseases: 1.05 (0.99, 1.11) - acute respiratory infections: 1.07 (0.97, 1.18) - COPD (chronic obstructive pulmonary disease): 1.06 (0.90, 1.25) - asthma: 1.09 (0.90, 1.33) - **(all) respiratory diseases (age ≤ 14 years): 1.11 (1.01, 1.22), *p* < 0.05 ^a^** - **Acute respiratory infections (age ≤ 14 years): 1.20 (1.04, 1.38), *p* < 0.05 ^a^** - asthma (age ≤ 14 years): 1.13 (0.91, 1.41)	[35]
South Africa	Cross-sectional study	Regression models expressing the association between a 24-h average indoor PM_2.5_ exposure and lung function outcomes, in terms of slope coefficient (95% CI): - PM_2.5_ concentration level and forced expiratory volume in 1s (FEV_1_): −0.60 (−1.23, 0.01) - **PM_2.5_ concentration level and forced vital capacity (FVC): −2.12 (−3.39, −0.85), *p* < 0.05 ^d^** - PM_2.5_ concentration level and FEV_1_/FVC: −1.42 (−4.85, 2.01)	[37]
Missouri (USA)	Cross-sectional study	Differences in the prevalence of diseases, between the two groups, in terms of significance: - *p* > 0.05 ^e^: ever told asthma; asthma attack in last 12 months; ever told have chronic obstructive pulmonary disease (COPD); nasal allergies in last 12 months; wheezing, cough, eye irritation, fatigue (tiredness), headaches, nausea, trouble sleeping in the last 12 months - **PM_2.5_ concentration level and forced vital capacity (FVC): −2.12 (−3.39, −0.85), *p* < 0.05 ^d^** - ***p* < 0.05 ^e^: other respiratory conditions (the most commonly reported included pneumonia, sleep-related disorders, and bronchitis)** - ***p* < 0.01 ^e^: attack of shortness of breath in the last 12 months**	[39]
China	Cross-sectional study	Students in non-exposure areas had significantly (*p* < 0.05 ^f^) higher levels of lysozyme, secretory immunoglobulin A (SIgA), and better lung capacity than students in exposed areas	[40]
North Carolina (USA)	Cross-sectional study	Symptoms associated to odour (odds ratio (OR) and 95% confidence interval (CI)): - *p* > 0.05 ^e^: ever told asthma; asthma attack in last 12 months; ever told have chronic obstructive pulmonary disease (COPD); nasal allergies in last 12 months; wheezing, cough, eye irritation, fatigue (tiredness), headaches, nausea, trouble sleeping in the last 12 months - **upper respiratory symptoms 3.9 (2.2, 7.0), *p* < 0.05 ^a^**	[38]
***Gastroenteritis***			
North Carolina (USA)	Cross-sectional study	Symptoms associated to odour (odds ratio (OR) and 95% confidence interval (CI)): - gastrointestinal symptoms 1.0 (0.4, 2.6)	[38]
***Mental and social health conditions***			
North Carolina (USA)	Cross-sectional study	Symptoms associated to odour (odds ratio (OR) and 95% confidence interval (CI)): - **alteration of daily activities: 9.0 (3.5, 23.5), *p* < 0.05 ^a^** - **negative mood states: 5.2 (2.8, 9.6), *p* < 0.05 ^a^** - positive mood states: 0.6 (0.2, 1.5)	[38]

^a^*p* < 0.05. Estimated in our systematic review on the basis of 95% Confidence Interval; ^b^ The sum of anomalies divided by the total proximal sum of births; ^c^ People living beyond the 2-km zone of all known landfill sites represented the reference population; ^d^ *p*< 0.05. Value from regression models. ^e^ *p*-value for test of equality; ^f^ Multiple linear regression models were conducted by the authors to determine the associations between health end points and air pollutants.

**Table 3:** Health outcomes associated with landfills.

Study Location	Study Design	Study Participants	Study Period	Exposure Source	Outcomes Investigated	Ref.
Italy	Cohort study (retrospective)	21,517 births in women (aged 15–49 years) residing within 4 km from an incinerator	Residents between 2003 and 2010	8 MSW incinerators operating in the Emilia Romagna region	Assessment of the effects of air emissions from MSW incinerators (simulated with a dispersion model) on reproductive outcomes ^a^	[45]
Italy	Cohort study (retrospective)	11,875 pregnancies with 1375 miscarriages from women (aged 15–24 years) residing within 4 km from a MSW incinerator	Residents between 2002 and 2006	7 MSW incinerators operating in the Emilia Romagna region	Assessment of the effects of air emissions from MSW incinerators (simulated with a dispersion model) on spontaneous abortions	[52]
France	Case-control study	Comparison of 304 infants with urinary tract birth defects with a control group of 226 infants randomly selected in the same region	Between 2001 and 2004	21 MSW incinerators active in the Rhone-Alps region	Association between the risk of urinary tract birth defects and living near MSW incinerators, using a model to predict the exposure to dioxins	[46]
Great Britain (UK)	Cohort study (retrospective)	1,025,064 births and 18,694 infant deaths in Great Britain. Incinerators emissions within 10 km were considered	Births and deaths between 2003 and 2010	22 MSW incinerators (operating between 2003 and 2010)	Associations between modelled ground-level particulate matter from incinerators emission within 10 km and selected reproductive/birth outcomes	[24]
Taiwan	Cohort study (retrospective)	6697 neonates assessed one year before the MSW incinerator started, and 6282 neonates assessed five years later incinerator opening	Neonates in 1991 and in 1997	The MSW incinerator of Taipei	The relationships between exposure to elevated PCDD/Fs concentration generated by a MSW incinerator (using a model), and various birth outcomes	[51]
Spain	Cohort study (perspective)	104 exposed subjects (living < 1 km from the MSW incinerator) and 97 non-exposed subjects (living > 3 km from the incinerator) were randomly selected. From 1999 one additional group (100 unexposed subjects, in Arenys de Mar, about 11 km from the incinerator) was selected	7 different campaigns were performed between 1995 and 2012	The MSW incinerator of Matarò (activated in 1995)	To monitor PCDD/Fs and PCBs levels in blood samples in the different exposed groups	[53]
England and Scotland (UK)	Cohort study (retrospective)	219,486 births, stillbirths, and terminations of pregnancy for foetal anomaly, in which 5154 were cases of congenital anomalies. Incinerators emissions within 10 km were considered	Birth and adverse birth outcomes between 2003 and 2010	10 MWIs in England and Scotland (operating between 2003 and 2010)	Associations between modelled ground-level particulate matter from incinerators emission within 10 km and selected reproductive/birth outcomes	[25]
Italy	Cohort study (retrospective)	31,347 residents within a 3.5 km radius of two incinerators	Residents between 1990 and 2003	An MSW incinerator and a hospital waste incinerator in Forlì	Health outcomes among people living close to incinerators (using a dispersion model for exposure assessment)	[44]
France	Case-control study	434 incident cases of invasive breast cancer diagnosed (case group) compared with 2170 controls randomly selected	Between 1996 and 2002 (cancer diagnosis in the case group).1999 (control group) ^b^	The MSW incinerator in Besançon	The association between dioxins emitted from a MSW incinerator (air exposure using a model) and invasive breast cancer risk among women residing in the area	[48]
Italy	Cohort study (retrospective)	Women residing or working near a MSW incinerator of Modena	Residents or workers between 2003 and 2006	The MSW incinerator of Modena	Rates of spontaneous abortion and prevalence of birth defects among women living or working near a MSW incinerator, modelling incinerator emissions exposure	[49]
Italy	Case-control study	Women (aged 16–44 years) residing near a MSW incinerators, assessing 228 cases of congenital anomalies	Birth defects between 1998 and 2006	The MSW incinerator of Reggio Emilia	The relationship between exposure to the emissions from an MSW incinerator and risk of birth defects, modelling incinerator emissions exposure	[50]
China	Cross-sectional study	82 children living near a MSW incinerator in China and 49 from a control area, both in Zhejiang Province	Samples collected in October 2013	A MSW incinerator in the Zhejiang Province	To monitor PCDD/F levels in blood in different exposed groups	[9]
China	Cross-sectional study	14 mothers living near a MSW incinerator (exposure area) and 18 mothers from a control area, both in Zhejiang Province	Samples collected in September and October 2013	A MSW incinerator in the Zhejiang Province	To monitor PCDD/Fs and PCBs in the breast milk of mothers in different exposed groups	[47]

^a^ The estimated annual average exposure to PM_10_ from incinerators in the study areas was 0.96 ng/m^3^ in 2003, decreasing to 0.26 ng/m^3^ in 2010 because of the improvements of the plant during the study period; ^b^ Some weaknesses in the study: controls were residents in 1999, whereas cases were diagnosed between 1996 and 2002, introducing a time lag in the sampling for some matched sets.

**Table 4:** Health outcomes associated with incinerators.

Study Location	Study Design	Main Findings (e.g., Estimated Risk, CI, *p*-Value)	Ref.
***Mortality***			
Italy	Cohort study (retrospective)	Associations between heavy metals concentration and mortality in the highest exposed group using the lowest exposure category as the reference (rate ratio (RR) and 95% CI): - all causes (men): 1.01 (0.86, 1.20) - **all causes (women): 1.12 (1.00, 1.27) ^a^** - >cardiovascular diseases (men): 0.98 (0.75, 1.29) - cardiovascular diseases (women): 1.32 (1.00, 1.72) - ischemic heart diseases (men): 0.79 (0.51, 1.22) - ischemic heart diseases (women): 1.14 (0.72, 1.82) - respiratory diseases (men): 1.01 (0.42, 2.45) - respiratory diseases (women): 0.53 (0.18, 1.56) - chronic pulmonary diseases (men): 0.53 (0.15, 1.86) - chronic pulmonary diseases (women): 0.27 (0.03, 2.06) Associations between heavy metals concentration and cancer mortality in the highest exposed group using the lowest exposure category as the reference (rate ratio (RR) and 95% CI): - all cancer (men): 0.85 (0.64, 1.12) - **all cancer (women): 1.47 (1.09, 1.99) ^a^** - stomach (men): 0.85 (0.35, 2.03) - stomach (women): 1.86 (0.73, 4.75) - colon rectum (men): 2.05 (0.92, 4.58) - colon rectum (women): 2.15 (0.86, 5.37) - liver (men): 0.27 (0.03, 2.18) - liver (women): 5.10 (0.94, 27.80) - larynx (men): no cases - larynx (women): no cases - lung (men): 0.91 (0.53, 1.57) - lung (women): 0.96 (0.31, 2.97) - soft tissue sarcoma (men): no cases - soft tissue sarcoma (women): no cases - breast (women): 2.00 (1.00, 3.99) - prostate (men): 1.57 (0.66, 3.74) - bladder (men): 1.48 (0.52, 4.22) - bladder (women): 3.06 (0.64, 14.70) - central nervous system (men): no cases - central nervous system (women): no cases - lymph. system (men): 0.42 (0.15, 1.23) - lymph. system (women): 1.78 (0.74, 4.25) - non-Hodgkin lymphoma (men): 0.52 (0.11, 2.45) - non-Hodgkin lymphoma (women): 2.03 (0.48, 8.67) - myeloma (men): no cases - myeloma (women): 4.28 (0.77, 23.80) - leukaemia (men): 0.67 (0.14, 3.16) - leukaemia (women): 1.31 (0.25, 6.95)	[44]
***Cancer***			
Italy	Cohort study (retrospective)	Associations between heavy metals concentration and cancer incidence in the highest exposed group using the lowest exposure category as the reference (Rate Ratio (RR) and 95% CI): - all cancer (men): 0.87 (0.72, 1.06) - all cancer (women): 0.90 (0.73, 1.11) - stomach (men): 1.24 (0.64, 2.40) - stomach (women): 1.09 (0.49, 2.44) - colon rectum (men): 1.00 (0.57, 1.75) - colon rectum (women): 1.33 (0.71, 2.48) - liver (men): 0.26 (0.03, 2.01) - liver (women): 0.94 (0.20, 4.53) - larynx (men): 0.15 (0.02, 1.14) - larynx (women): 1.60 (0.15, 17.64) - lung (men): 0.96 (0.61, 1.52) - lung (women): 0.81 (0.27, 2.42) - soft tissue sarcoma (men): 0.84 (0.09, 8.06) - soft tissue sarcoma (women): no cases - breast (women): 0.76 (0.51, 1.13) - prostate (men): 1.27 (0.82, 1.99) - bladder (men): 0.78 (0.43, 1.42) - bladder (women): 2.30 (0.73, 7.24) - central nervous system (men): 1.35 (0.34, 5.39) - central nervous system (women): no cases - lymph. system (men): 0.70 (0.38, 1.28) - lymph. system (women): 1.23 (0.65, 2.33) - non-Hodgkin lymphoma (men): 0.59 (0.23, 1.57) - non-Hodgkin lymphoma (women): 1.06 (0.39, 2.93) - myeloma (men): 0.61 (0.17, 2.13) - myeloma (women): 0.95 (0.26, 3.45) - leukaemia (men): 1.01 (0.36, 2.84) - leukaemia (women): 1.23 (0.33, 4.62)	[44]
France	Case-control study	Odds ratio (OR) of invasive breast cancer by age bands and dioxin exposure categories (comparing very low with high exposure) (95% CI): - women aged 20–59 years: 0.88 (0.43, 1.79) - women aged 60 years and over: 0.31 (0.08, 0.89)	[48]
***Adverse birth and neonatal outcomes***			
Italy	Cohort study (retrospective)	Associations between modelled exposure levels to PM_10_ from the incinerators and reproductive outcomes, for the highest versus the lowest quintile exposure (odds ratio (OR), 95% confidence interval and significance): - **preterm births: 1.30 (1.08, 1.57) ^b^, *p* < 0.05 ^c^; 1.44 (1.11, 1.85) ^d^, *p* < 0.05 ^c^** - sex ratio: 0.91 (0.83, 0.99) ^b^; 0.88 (0.78, 0.99) - multiple births: 0.87 (0.57, 1.33) ^b^; 1.12 (0.60, 2.08) ^d^ - small for gestational age (SGA): 1.11 (0.96, 1.28) ^b^; 1.06 (0.87, 1.29) ^d^	[45]
Italy	Cohort study (retrospective)	Associations between modelled exposure levels to PM_10_ from the incinerators and miscarriages, for the highest versus the lowest quintile exposure (adjusted odds ratio (OR), 95% confidence interval and significance p): - spontaneous abortions: 1.29 (0.97, 1.72) ^e^	[52]
Italy	Cohort study (retrospective)	Associations between modelled exposure levels of pollutants from the incinerator and reproductive outcomes, in terms of Relative Risk computed as the ratio between observed and expected incidence, (95% confidence interval): - Spontaneous abortion: - residents from both areas A and B 1.00 (0.65, 1.48) - area A residents (highest exposure): 0.87 (0.22, 2.38) - area B residents (intermediate exposure): 1.03 (0.64, 1.56) - workers from both areas A and B: 1.04 (0.38, 2.30) - area A workers: 0.00 (0.00, 1.46) - area B workers: 1.81 (0.66, 4.02) - Spontaneous abortion: - residents from both areas A and B: 0.64 (0.20, 1.55) - area A residents: 0.00 (0.00, 4.41) - area B residents: 0.72 (0.23, 1.75) - workers from both areas A and B: 2.26 (0.57, 6.14) - area A workers: 2.22 (0.37, 7.34) - area B workers: 2.27 (0.11, 11.21)	[49]
Great Britain (UK)	Cohort study (retrospective)	Associations between modelled exposure levels of pollutants from the incinerator and reproductive outcomes (adjusted OR and 95% CI): - stillbirths ^f^: 0.99 (0.97, 1.00) - stillbirths ^g^: 1.00 (0.99, 1.02) - neonatal mortality (pregnancy exposure) ^f^: 0.99 (0.96, 1.02) - neonatal mortality (pregnancy exposure) ^g^: 1.01 (1.00, 1.03) - post-neonatal mortality (pregnancy exposure) ^f^: 1.02 (0.96, 1.07) - post-neonatal mortality (pregnancy exposure) ^g^: 0.99 (0.97, 1.02) - post-neonatal mortality (birth to death of case exposure) ^f^: 1.01 (0.98, 1.04) - multiple births ^f^: 0.99 (0.99, 1.00) - multiple births ^g^: 1.00 (0.99, 1.00) - sex ratio ^f^: 1.00 (1.00, 1.00) - sex ratio ^g^: 1.00 (1.00, 1.00) - preterm delivery ^f^: 0.99 (0.97, 1.01) - preterm delivery ^g^: 1.00 (0.99, 1.00) - terms small for gestational age (SGA) ^f^: 0.99 (0.98, 1.00) - terms SGA ^g^: 1.00 (0.99, 1.01)	[24]
England and Scotland (UK)	Cohort study (retrospective)	Adjusted odds ratio (OR) (95% CI): - all congenital anomalies ^f^: 1.00 (0.98, 1.02) - all congenital anomalies ^g^: 1.02 (1.00, 1.04) - all congenital anomalies excluding chromosomal ^f^: 0.99 (0.97, 1.01) - all congenital anomalies excluding chromosomal ^g^: 1.02 (1.00, 1.04) - nervous system ^f^: 0.97 (0.92, 1.02) - nervous system ^g^: 0.97 (0.93, 1.02) - congenital heart defects ^f^: 0.99 (0.93, 1.05) - **congenital heart defects ^g^: 1.04 (1.01, 1.08), *p* < 0.05 ^h^** - abdominal wall defects ^f^: 1.00 (0.92, 1.08) - abdominal wall defects ^g^: 1.00 (0.94, 1.07) - oro-facial clefts ^f^: 1.00 (0.94, 1.07) - oro-facial clefts ^g^: 0.99 (0.94, 1.05) - limb defects ^f^: 1.01 (0.94, 1.08) - limb defects ^g^: 1.02 (0.97, 1.08) - digestive system ^f^: 1.00 (0.92, 1.09) - digestive system ^g^: 1.00 (0.95, 1.06) - urinary system ^f^: 1.00 (0.94, 1.07) - urinary system ^g^: 1.02 (0.97, 1.06) - genital system ^f^: 1.03 (0.95, 1.13) - **genital system ^g^: 1.07 (1.02, 1.12), *p* < 0.05 ^h^** - neural tube defects (from congenital anomaly sub-groups (CAS)) ^f^: 1.00 (0.92, 1.07) - neural tube defects (from CAS) ^g^: 0.97 (0.91, 1.03) - severe congenital heart defects (from CAS) ^f^: 1.03 (0.97, 1.10) - severe congenital heart defects (from CAS) ^g^: 1.02 (0.97, 1.07) - gastroschisis (from CAS) ^f^: 1.04 (0.94, 1.15) - gastroschisis (from CAS) ^g^: 0.97 (0.89, 1.05) - cleft palate (from CAS) ^f^: 1.02 (0.92, 1.13) - cleft palate (from CAS) ^g^: 0.98 (0.90, 1.06) - cleft lip with or without cleft palate (from CAS) ^f^: 1.00 (0.93, 1.08) - cleft lip with or without cleft palate (from CAS) ^g^: 1.00 (0.94, 1.07) - limb reduction defects (from CAS) ^f^: 1.02 (0.91, 1.14) - limb reduction defects (from CAS) ^g^: 0.98 (0.90, 1.08) - oesophageal atresia (from CAS) ^f^: 1.04 (0.88, 1.22) - oesophageal atresia (from CAS) ^g^: 0.92 (0.80, 1.05) - anomalies of the renal system (from CAS) ^f^: 1.02 (0.95, 1.10) - anomalies of the renal system (from CAS) ^g^: 1.00 (0.93, 1.07) - obstructive defects of renal pelvis (from CAS) ^f^: 0.97 (0.90, 1.04) - obstructive defects of renal pelvis (from CAS) ^g^: 1.03 (0.97, 1.10) - hypospadias (from CAS) ^f^: 1.00 (0.90, 1.12) - **hypospadias (from CAS) ^g^: 1.07 (1.01, 1.12), p < 0.05^h^**	[25]
Taiwan	Cohort study (retrospective)	Difference of birth outcomes between higher exposure and control areas in 1997 (adjusted OR and 95% CI): - birth weight: 1.06 (0.71, 1.57) - gestation weeks, in 1997: 1.22 (0.97, 1.52) - gender, in 1997: 0.90 (0.78, 1.05)	[51]
Italy	Case-control study	Prevalence (odds ratio) for congenital anomalies according to maternal exposure to air emissions from the incinerator (95% confidence interval), with low exposure area as reference: All congenital anomalies: - area B (medium exposure) ^i^: 1.55 (0.67, 3.56) - area B ^j^: 1.10 (0.39, 3.06) - area B ^k^: 3.17 (0.65, 15.46) - area C (high exposure) ^i^: 0.67 (0.25, 1.77) - area C ^j^: 0.41 (0.11, 1.61) - area C ^k^: 1.30 (0.29, 5.82) Cardiovascular anomalies: - area B ^i^: 0.94 (0.27, 3.31) - area C ^i^: 0.58 (0.14, 2.45) - area B ^j^: 0.59 (0.14, 2.49)	[50]
France	Case-control study	Risk of urinary tract birth defects, in terms of OR (with 95% CI), for not exposed group versus exposed above the median: - considering atmospheric dioxins: 2.84 (1.32, 6.09) ^h^ - considering dioxin deposits: 2.95 (1.47, 5.92) ^h^ - considering metals: 0.73 (0.45, 1.19) - considering consumption of local food and dioxin deposits: 1.88 (0.55, 6.35)	[46]
***Cardiovascular diseases***			
Italy	Cohort study (retrospective)	Associations between heavy metals concentration and hospitalization for specific causes in the highest exposed group using the lowest exposure category as the reference (rate ratio (RR) and 95% CI): - acute myocardic infarction (men): 0.81 (0.51, 1.28) - acute myocardic infarction (women): 1.40 (0.66, 2.98) - chronic heart failure (men): 0.78 (0.46, 1.33) - chronic heart failure (women): 1.48 (0.90, 2.46)	[44]
***Respiratory conditions***			
Italy	Cohort study (retrospective)	Associations between heavy metals concentration and hospitalization for specific causes in the highest exposed group using the lowest exposure category as the reference (rate ratio (RR) and 95% CI): - chronic obstructive pulmonary disease (men): 1.43 (0.89, 2.31) - chronic obstructive pulmonary disease (women): 0.63 (0.35, 1.14) - acute respiratory diseases (men): 0.89 (0.63, 1.27) - acute respiratory diseases (women): 1.29 (0.94, 1.78) - asthma (men): 1.16 (0.36, 3.71) - asthma (women): 1.01 (0.40, 2.55)	[44]
***Human biomonitoring**^**l, m, n**^*			
China	Cross-sectional study	Blood PCDD/F levels comparing exposed group with control group: - TEQΣPCDD/Fs: **0.40 vs. 0.28 pg TEQ/g wet weight, *p* < 0.05 ^o^**	[9]
China	Cross-sectional study	PCDD/Fs and PCBs levels in breast milk comparing exposed and control groups: - TEQ (PCDD/Fs + DL-PCBs): **0.28 vs. 0.16 pg TEQ/g wet weight, *p* < 0.05 ^p^** Mean EDI level in infants comparing exposed and control groups: **22.0 vs. 13.0 pg TEQ/kg bw day, *p* < 0.05 ^p^**	[47]
Spain	Cohort study (perspective)	Concentrations of PCDD/Fs, expressed as pg TEQ/g fat in whole blood samples in exposed/non-exposed (Matarò)/non-exposed (Arenys de Mar): - 1995: 13.0/13.1/Not Measured (NM) - 1997: 15.9/16.4/NM - 1999: 17.8/18.1/18.7 - 2002: 15.1/18.2/16.02 - 2005: 11.7/12.3/17.9 - 2008: 14.6/12.6/14.5 - 2012: 12.9/13.3/12.5	[53]

^a^ The authors indicated the level of significance only when *p*-value was lower than 0.05. ^b^ period 2003–2010; ^c^ *p* < 0.05. Test conducted by the authors for trend across categories of exposure to incinerator emissions; ^d^ period 2007–2010; ^e^ The authors reported a *p*-value of 0.042, for testing the trend of groups 1 and 5 (the highest versus the lowest quintile). It can be noted a significant trend for increases in spontaneous abortions with greater PM exposure. ^f^ Per doubling of P_M10;_^g^ Proximity to the nearest MWI, calculated as a continuous measure of linear distance (km); ^h^ *p* < 0.05. Estimated in our systematic review on the basis of 95% Confidence Interval; ^i^ Entire study period; ^j^ Operation period: from December 1 1998 to October 31 2002 and from April 1 2006 to December 31 2006; ^k^ Shut-down period: from 1 February 2003 to 31 December 2005; ^l^ In terms of dioxins, whose long-term exposure increases the risk of cancer and other negative health outcomes including reproductive, developmental and neurodevelopmental effects [54,55]; ^m^ Values expressed in terms of Toxic Equivalence (TEQ) were assessed. Indeed, TEQs are calculated values that allow to compare the toxicity of different combinations of dioxins and dioxin-like compounds; in order to calculate a TEQ, a toxic equivalent factor (TEF) is assigned to each member of the dioxin and dioxin-like compounds category. TEFs have been established through international agreements and currently range from 1 to 0.0001 [56]; ^n^ EFSA et al. [57] considered a threshold value in serum of 7.0 pg/g fat. Furthermore, they established a Tolerable Weekly Intake (TWI) of 2 pg TEQ/kg bw per week. WHO [55] indicates a provisional tolerable intake of 70 pg/kg bw per month for PCDDs, PCDFs and coplanar PCBs expressed as TEFs. It has to be noted that although several studies showed a positive association with cancer, there was no clear dose–response relationship between exposure and cancer development [57]; at the same time, WHO [55] noted since dioxins induce tumors and likely other effects via a receptor-mediated mechanism, tolerable intake guidance based on non-cancer end-points observed at lower doses is considered protective for carcinogenicity. ^o^ *p* < 0.05. When data fit the normal distribution, two independent sample *t*-tests were performed by the authors to compare the mean levels of the two groups. Otherwise, the Mann–Whitney U test was performed. ^p^ *p* < 0.05. If the data fitted the normal distribution, two independent sample *t*-tests were performed by the authors to compare the mean levels of the two groups. Otherwise, the non-parametric test was performed.

**Table 5:** Dumpsites and open burning—methodology characterizing each research.

Study Location	Study Design	Study Participants	Study Period	Exposure Source	Outcomes Investigated	Ref.
Swaziland	Cross-sectional study	78 residents in an area very close to a dumpsite and 39 people closer (<200 m) and 39 further away (>200 m) from the dumpsite	The authors did not specify the period of the questionnaires	A dumpsite in Manzini city	To determine the health effects of a dumpsite on the surrounding human settlement through self-administered questionnaires	[20]
Nigeria	Cross-sectional study	100 household residents within 250 m radius of a dumpsite and 100 household residents between 250–500 metres from the same dumpsite	Data collected from 23 October 2015 to 5 November 2015	A dumpsite in Lagos	To determine the health effects of a dumpsite on the surrounding human population through self-administered questionnaires	[61]
Brazil	Cohort study (retrospective)	People living within 2 km from the 15 landfills in the municipality of São Paulo	Between 1998 and 2002.	The 15 solid waste landfill sites within the municipality of São Paulo (all, except one, were controlled dumpsite with no waterproof layer at the bottom)	To evaluate the association between living close to a controlled dumpsite and occurrences of deaths for cancer or congenital malformations	[59]
Alaska	Cohort study (retrospective)	10,073 infants born in 197 villages close to dumpsites (ranked in high, intermediate, and low hazard)	Infants born between 1997 and 2001	197 dumpsites	To evaluate adverse birth outcomes (low and very low birth weight, preterm birth, and intrauterine growth restriction (IUGR)) in infants born close to dumpsites	[58]
Alaska	Cohort study (retrospective)	10,360 infants born in 197 villages close to dumpsites (ranked in higher and lower hazard)	Infants born between 1997 and 2001	197 dumpsites	To evaluate the rates of adverse pregnancy outcomes as foetal death, neonatal death, congenital anomalies, close to dumpsites	[60]
Sierra Leone	Cross-sectional study	398 residents nearby (<50 m) and 233 residents further away (>50 m) a dumpsite	The authors did not specify the period of the questionnaires	A dumpsites in Freetown	To determine the health effects of a dumpsite on the surrounding human population through self-administered questionnaires	[62]
Ghana	Cross-sectional study	150 residents in a community nearby dumpsites, comparing three distances between people and disposal sites: (a) less than 5 min, (b) 5–10 min, (c) 11–15 min ^a^	The authors did not specify the period of the questionnaires	A dumpsite in the Ashanti Region	To determine the health effects of dumpsites on the surrounding human population through self-administered questionnaires	[63]

^a^ The authors did not write how many of the people interviewed lived in zone (a), (b), (c).

**Table 6:** Health outcomes associated with dumpsites and open burning.

Study Location	Study Design	Main Findings	Ref.
***Mortality***			
Brazil	Cohort study (retrospective)	Standardized mortality ratios (SMRs) for areas of 2 km around the solid waste landfill sites (95% CI): - bladder cancer: 0.98 (0.79, 1.21) - liver cancer: 1.00 (0.86, 1.16) - leukaemia in adults: 0.92 (0.77, 1.10) - leukaemia in children: 0.84 (0.54, 1.31) Standardized mortality ratios (SMRs) for areas of 2 km around the solid waste landfill sites (95% CI): - congenital malformation: 0.86 (0.72, 1.03)	[59]
***Adverse birth and neonatal outcomes***			
Alaska	Cohort study (retrospective)	Adjusted odds ratios (95% CI) describing the relations between low and high hazard exposure categories and incidence of low and very low birth weight, preterm birth, and intrauterine growth retardation: - **low birth weight: 2.06 (1.28, 3.32), *p* < 0.05 ^a^** - **low birth weight adjusted for gestation: 2.20 (1.26, 3.85), *p* < 0.05 ^a^** - very low birth weight: 1.17 (0.37, 3.67) - preterm birth: 1.24 (0.89, 1.74) - **intrauterine growth retardation: 3.98 (1.93, 8.21), *p* < 0.05 ^a^**	[58]
Alaska	Cohort study (retrospective)	Adjusted rate ratios (95% CI) describing the relationships between lower and higher hazard exposure categories and incidence of foetal and neonatal death and congenital anomalies: - all deaths: 0.65 (0.34, 1.27) - foetal deaths: 0.75 (0.28, 1.99) - neonatal deaths: 0.55 (0.22, 1.38) - all congenital anomalies (CA), (listed separately in the categories below): 1.37 (0.92, 2.04) - central nervous system CA: 2.36 (0.37, 14.71) - circulatory/respiratory CA: 1.42 (0.39, 5.42) - gastrointestinal CA: 0.58 (0.14, 2.40) - urogenital CA: 2.71 (0.67, 10.95) - musculoskeletal/integumental CA: 1.61 (0.79, 3.29) - others CA: 1.38 (0.77, 2.39) - multiple CA: 1.33 (0.34, 5.20)	[60]
***Gastroenteritis***			
Swaziland	Cross-sectional study	Diseases which affected residents: - diarrhoea: 16% of closer residents vs. 5% of further away residents Reasons for hospitalization among the interviewed: - diarrhoea: 16% of closer residents vs. 26% of further away residents - cholera: 12% of closer residents vs. 0% of further away residents	[20]
Nigeria	Cross-sectional study	Diseases which affected residents ^b^: - cholera and diarrhoea: 10 closer households vs. 5 further away households reported 1–2 cases; 0 closer households vs. 0 further away households reported 3–4 cases; 0 closer households vs. 0 further away households reported at least 5 cases	[61]
Sierra Leone	Cross-sectional study	Diseases which affected residents ^c^: - diarrhoea: about 10% of closer residents vs. about 12% of further away residents - cholera: about 11% of closer residents vs. about 15% of further away residents	[62]
Ghana	Cross-sectional study	Diseases which affected residents ^d^: - cholera: (a) 67%; (b) 33%; (c) 0% (out of a total of 6 people affected) - typhoid fever: (a) 75%; (b) 25%; (c) 0% (out of a total of 12 people affected)	[63]
***Vector-borne diseases***			
Swaziland	Cross-sectional study	Diseases which affected residents: - malaria: 36% of closer residents vs. 13% of further away residents Reasons for hospitalization among the interviewed: - malaria: 44% of closer residents vs. 18% of further away residents	[20]
Nigeria	Cross-sectional study	Diseases which affected residents ^b^: - malaria: 20 closer households vs. 24 further away households reported 1–2 cases; 4 closer households vs. 8 further away households reported 3–4 cases; 0 closer households vs. 1 further away households reported at least 5 cases	[61]
Sierra Leone	Cross-sectional study	Diseases which affected residents ^c^: - malaria: 40% of closer residents vs. 35% of further away residents	[62]
Ghana	Cross-sectional study	Diseases which affected residents ^d^: - malaria: (a) 73%; (b) 25%; (c) 2% (out of a total of 103 people affected)	[63]

^a^*p* < 0.05. The authors indicated the *p*-value when it was lower than 0.05; ^b^ The authors categorized counts of reported cases into groups for each health outcome and then used a chi-square test to test for differences. No significant differences were found; ^c^ The % is an approximate value taken from a figure in the article; ^d^ Comparing three temporal distances between people and disposal sites: (a) less than 5 min, (b) 5–10 min, (c) 11–15 min.

**Table 7:** Evidence to develop health outcomes among residents living nearby landfills, incinerators, and dumpsites/open burning.

Heading	Mortality	Cancer	Adverse Birth and Neonatal Outcomes	Cardiovascular Diseases	Respiratory Conditions	Gastroenteritis	Vector-Borne Diseases	Mental Health Conditions	Human Biomonitoring ^a^
Landfills ^b^	+ (1)	0	+ (4)	− (1)	+ (5)	− (1)	0	+ (1)	0
Incinerators ^b^	+ (1)	− (2)	+ (8)	− (1)	− (1)	0	0	0	+ (3)
Dumpsites and Open Burning ^b^	− (1)	0	+ (2)	0	0	− (4)	− (4)	0	0

^a^ Human biomonitoring studies measured dioxins, whose long-term exposure increases the risk of cancer and other negative health outcomes including reproductive, developmental, and neurodevelopmental effects [54,55]; ^b^ Strength of evidence: 0: no studies; (−): No evidence of increased risk; (+): Some evidence of increased risk; (++): Strong evidence of increased risk. The number in parentheses beside each symbol represents the total number of studies that assessed each health outcome (which are reported in detail in Table 2, Table 4 and Table 6). Although the evidence for some outcomes was mixed, this number includes all the available studies, including both studies finding evidence and studies finding no evidence of an increased risk for each outcome.

## Supplementary Materials

The following are available online at https://www.mdpi.com/article/10.3390/ijerph18084331/s1: List of the studies screened (excluding duplicates)—in alphabetic order.
