# Supplementary material for: Municipal Solid Waste Management and Adverse Health Outcomes: A Systematic Review
Source: Int J Environ Res Public Health. 2021 Apr 19;18(8):4331. doi: 10.3390/ijerph18084331 (PMC8072713; doi:10.3390/ijerph18084331)
Supplement: Supplementary file 1 [file ijerph-18-04331-s001.zip › ijerph-1153581-supplementary.pdf]

Municipal Solid Waste Management and Adverse Health Outcomes: A systematic review

Giovanni Vinti, Valerie Bauza, Thomas Clasen, Kate Medlicott, Terry Tudor, Christian Zurbrügg, Mentore Vaccari

Supplementary material

List of the studies screened (excluding duplicates)—in alphabetic order

1. Aatamila, M, Verkasalo, PK, Korhonen, MJ, Suominen, AL, Hirvonen, MR, Viluksela, MK, Nevalainen, A. Odour annoyance and physical symptoms among residents living near waste treatment centres. *Environmental research* **2011**, 111 (1), 164–170.
2. Abd El–Wahab, EW, Eassa, SM. Seroprevalence of HBV among Egyptian municipal solid waste workers. *Heliyon* **2019**, 5 (6), e01873.
3. Abd El–Wahab, EW, Eassa, SM, Lotfi, SE, El Masry, SA, Shatat, HZ, Kotkat, AM. Adverse health problems among municipality workers in Alexandria (Egypt). *International journal of preventive medicine* **2014**, 5 (5), 545–556.
4. Abdou, MH. Health impacts on workers in landfill in Jeddah City, Saudi Arabia. *Journal of the Egyptian Public Health Association* **2007**, 82(3–4), 319–329.
5. Abou–Elwafa, HS, El–Bestar, SF, El–Gilany, AH, Awad, E. Musculoskeletal disorders among municipal solid waste collectors in Mansoura, Egypt: a cross–sectional study. *BMJ open* **2012**, 2, e001338.
6. Abul, S. Environmental and health impact of solid waste disposal at Mangwaneni dumpsite in Manzini: Swaziland. *Journal of Sustainable Development in Africa* **2010**, 12, 7.
7. Achudume, AC, Olawale, JT. Microbial pathogens of public health significance in waste dumps and common sites. *Journal of Environmental Biology* **2007**, 28 (1) 151–154.
8. Ahamad, A, Raju, NJ, Madhav, S, Gossel, W, Wycisk, P. Impact of non–engineered Bhalswa landfill on groundwater from quaternary alluvium in Yamuna flood plain and potential human health risk, New Delhi, India. *Quaternary International* **2019**, 507, 352–369.
9. Akpeimeh, GF, Fletcher, LA, Evans, BE. Exposure to bioaerosols at open dumpsites: A case study of bioaerosols exposure from activities at Olusosun open dumpsite, Lagos Nigeria. *Waste Management* **2019**, 89. 37–47.
10. Ala, A, Stanca, CM, Bu–Ghanim, M, Ahmado, I, Branch, AD, Schiano, TD, Odin, JA, Bach, N. (2006). Increased prevalence of primary biliary cirrhosis near Superfund toxic waste sites. *Hepatology* **2006**, 43 (3), 525–531.
11. Alam, P, Ahmade, K. Impact of solid waste on health and the environment. *International Journal of Sustainable Development and Green Economics* **2013**, 2, 2.

12. Alburquerque, JA, ì de la Fuente, C, Ferrer–Costa, A, Carrasco, L, Cegarra, J, Abad, M, Bernal, MP. Assessment of the fertiliser potential of digestates from farm and agroindustrial residues. *Biomass Bioenergy* **2012**, 40 (2012), pp. 181–189.
13. Al–Delaimy, WK, Larsen, CW, Pezzoli, K. Differences in Health Symptoms among Residents Living Near Illegal Dump Sites in Los Laureles Canyon, Tijuana, Mexico: A Cross Sectional Survey. *Int. J. Environ. Res. Public Health* **2014**, 11, 9532–9552.
14. Almazán–Casali, S, Alfaro, JF, Sikra, S. Exploring household willingness to participate in solid waste collection services in Liberia. *Habitat International* **2019**, 84, 57–64.
15. Amegah, A. K., Jaakkola, J. J., Quansah, R., Norgbe, G. K., Dzodzomenyo, M. Cooking fuel choices and garbage burning practices as determinants of birth weight: a cross–sectional study in Accra, Ghana. *Environmental health* **2012**, 11, 78.
16. Ashworth, DC, Elliott, P, Toledano, MB. Waste incineration and adverse birth and neonatal outcomes: a systematic review. *Environment International* **2014**, 69, 120–132.
17. Asibey, MO, Amponsah, O, Yeboah, V. Solid waste management in informal urban neighbourhoods. Occupational safety and health practices among tricycle operators in Kumasi, Ghana. *International journal of environmental health research* **2019**, 29(6), 702–717.
18. Athanasiou, M, Makrynos, G, Dounias, G. (2010). Respiratory health of municipal solid waste workers. *Occupational medicine* **2010**, 60(8), 618–623.
19. Auler, F, Nakashima, AT, Cuman, RK. Health conditions of recyclable waste pickers. *Journal of community health* **2014**, 39 (1), 17–22.
20. Babs–Shomoye, F, Kabir, R. Health Effects of Solid Waste Disposal at a Dumpsite on the Surrounding Human Settlements. *Journal of Public Health in Developing Countries* **2016**, 2 (3), 268–275.
21. Bagge, E. Hygiene Aspects of the Biogas Process with Emphasis on Spore–Forming Bacteria. PhD Thesis. Swedish University of Agricultural Sciences Uppsala, 2009.
22. Bastian, L, Yano, J, Hirai, Y, Sakai, S. Behavior of PCDD/Fs during open burning of municipal solid waste in open dumping sites. *J Mater Cycles Waste Manag* **2013**, 15, 229–241.
23. Beníšek, M, Kukučka, P, Mariani, G, Suurkuusk, G, Gawlik, BM, Locoro, G, Giesy, JP, Bláha, L. Dioxins and dioxin–like compounds in composts and digestates from European countries as determined by the in vitro bioassay and chemical analysis. *Chemosphere* **2015**, 122, 168–175.
24. Black, M, Karki, J, Lee, A, Makai, P, Baral, YR, Kritsotakis, EI, Bernier, A, Fossier Heckmann, A. The health risks of informal waste workers in the Kathmandu Valley: a cross–sectional survey. *Public health* **2019**, 166, 10–18.
25. Bleck, D, Wettberg, W. Waste collection in developing countries. Tackling occupational safety and health hazards at their source. *Waste management* **2012**, 32 (11), 2009–2017.

26. Boberg, E, Lessner, L, Carpenter, DO. The role of residence near hazardous waste sites containing benzene in the development of hematologic cancers in upstate New York. *International journal of occupational medicine and environmental h*ealth **2011**, 24 (4), 327–338.
27. Bogale, D, Kumie, A, Tefera, W. Assessment of occupational injuries among Addis Ababa city municipal solid waste collectors: a cross–sectional study. *BMC public health* **2014**, 14, 169.
28. Bray, L, Digka, N, Tsangaris, C, Camedda, A, Gambaiani, D, de Lucia, G A, Matiddi, M, Miaud, C, Palazzo, L, Pérez–Del–Olmo, A, Raga, JA, Silvestri, C, Kaberi, H. Determining suitable fish to monitor plastic ingestion trends in the Mediterranean Sea. *Environmental pollution* **2019**, 247, 1071–1077.
29. Breitenmoser, L, Gross, T, Huesch, R, Rau, J, Dhar, H, Kumar, S, Hugi, C, Wintgens, T. Anaerobic digestion of biowastes in India: Opportunities, challenges and research needs. *Journal of environmental management* **2019**, 236, 396–412.
30. Brennecke, D, Duarte, B, Paiva, F, Cacador, I, Canning–Clode, J. Microplastics as vectors for heavy metal contamination from the marine environment. *Estuar. Coast. Shelf Sci.* **2016**, 178, 189–195.
31. Breza–Boruta, B. The assessment of airborne bacterial and fungal contamination emitted by a municipal landfill site in Northern Poland. *Atmospheric Pollution Research* **2016**, 7, 1043–1052.
32. Briggs, DJ. A framework for integrated environmental health impact assessment of systemic risks. *Environmental health* **2008**, 7, 61.
33. Brinton, WF, Storms, P, Blewett, TC. Occurrence and levels of fecal indicators and pathogenic bacteria in market–ready recycled organic matter composts. *Journal of Food Protection* **2009**, 72 (2), 332–339.
34. Bünger, J, Schappler–Scheele, B, Hilgers, R, Hallier, E. A 5–year follow–up study on respiratory disorders and lung function in workers exposed to organic dust from composting plants. *International archives of occupational and environmental health* **2007**, 80 (4), 306–312.
35. Butt, TE, Gouda, HM, Baloch, MI, Paul, P, Javadi, AA, Alam, A. Literature review of baseline study for risk analysis – the landfill leachate case. *Environment international* **2014**, 63, 149–162.
36. Candela, S, Bonvicini, L, Ranzi, A, Baldacchini, F, Broccoli, S, Cordioli, M, Carretta, E, Luberto, F, Angelini, P, Evangelista, A, Marzaroli, P, Giorgi Rossi, P, Forastiere, F. Exposure to emissions from municipal solid waste incinerators and miscarriages: a multisite study of the MONITER Project. *Environment International* **2015**, 78, 51–60.
37. Candela, S, Ranzi, A, Bonvicini, L, Baldacchini, F, Marzaroli, P, Evangelista, A, Luberto, F, Carretta, E, Angelini, P, Sterrantino, AF, Broccoli, S, Cordioli, M, Ancona, C,

Forastiere, F. Air pollution from incinerators and reproductive outcomes: a multisite study. *Epidemiology* **2013**, 24 (6), 863–870.

38. Caravanos, J, Chatham–Stephens, K, Ericson, B, Landrigan, PJ., Fuller, R. The burden of disease from pediatric lead exposure at hazardous waste sites in 7 Asian countries. *Environmental research* **2013**, 120, 119–125.
39. Carpenter, DO, Ma, J, Lessner, L. Asthma and infectious respiratory disease in relation to residence near hazardous waste sites. *Annals of the New York Academy of Sciences* **2008**, 1140, 201–208.
40. Castilla, EE, Mastroiacovo, P, López–Camelo, JS, Saldarriaga, W, Isaza, C, Orioli, IM. Sirenomelia and cyclopia cluster in Cali, Colombia. American journal of medical genetics. Part A **2008**, 146A (20), 2626–2636.
41. Chatham–Stephens, K, Caravanos, J, Ericson, B, Landrigan, P, Fuller, R. The pediatric burden of disease from lead exposure at toxic waste sites in low and middle income countries. *Environmental research* **2014**, 132, 379–383.
42. Cheng, Z, Sun, Z, Zhu, S, Lou, Z, Zhu, N, Feng, L. The identification and health risk assessment of odor emissions from waste landfilling and composting. *Science of the Total Environment* **2019**, 649, 1038–1044.
43. Cocarta, DM, Rada, EC, Ragazzi, M, Badea, A, Apostol, T. A contribution for a correct vision of health impact from municipal solid waste treatments. *Environmental Technology* **2009**, 30 (9), 963–968.
44. Coelho, APF, Beck, CLC, Fernandes, MNS, Freitas, NQ, Prestes, FC, Tonel, JZ. Women waste pickers: living conditions, work, and health. *Revista Gaúcha de Enfermagem* **2016**, 37 (3), e57321.
45. Coelho, APF, Beck, CLC, Fernandes, MNS, Silva, RM, Reis, DAM. Organization of the work in a recycling cooperative: implications for the health of female waste pickers. *Cogitare Enferm* **2016**, 21 (1), 01–09.
46. Cointreau, S., 2006. Occupational and environmental health issues of solid waste management: special emphasis on middle and lower–income countries. The World Bank, Urban Solid Waste Management, Washington, DC.
47. Cordier, S, Lehébel, A, Amar, E, Anzivino–Viricel, L, Hours, M, Monfort, C, Chevrier, C, Chiron, M, Robert–Gnansia, E. Maternal residence near municipal waste incinerators and the risk of urinary tract birth defects. *Occupational and environmental medicine* **2010**, 67 (7), 493–9.
48. Cordioli, M, Ranzi, A, De Leo, GA, Lauriola, P. A review of exposure assessment methods in epidemiological studies on incinerators. *Journal of environmental and public health* **2013**, 2013, 129470.
49. Cruvinel, V, Marques, CP, Cardoso, V, Novaes, M, Araújo, WN, Angulo–Tuesta, A, Escalda, P, Galato, D, Brito, P., da Silva, EN. Health conditions and occupational risks in

a novel group: waste pickers in the largest open garbage dump in Latin America. *BMC public health* **2019**, 19 (1), 581.
50. Cunningham, RN, Simpson, CD, Keifer, MC. Hazards faced by informal recyclers in the squatter communities of Asunción, Paraguay. *International journal of occupational and environmental health* **2012**, 18 (3), 181–187.
51. Das, B, Bhave, PV, Sapkota, A, Byanju, RM. Estimating emissions from open burning of municipal solid waste in municipalities of Nepal. *Waste Management* **2018**, 79, 481–490.
52. da Silva, MC, Fassa, AG, Kriebel, D. Musculoskeletal pain in ragpickers in a southern city in Brazil. *American journal of industrial medicine* **2006**, 49 (5), 327–336.
53. Davoli, E, Fattore, E, Paiano, V, Colombo, A, Palmiotto, M, Rossi, AN, Il Grande, M, Fanelli, R. Waste Management Health Risk Assessment: A Case Study of a Solid Waste Landfill in South Italy. *Waste Management* **2010**, 30, 1608–1613.
54. De, S, Debnath, B. Prevalence of Health Hazards Associated with Solid Waste Disposal–A Case Study of Kolkata, India. Proceedings of the International Conference on Solid Waste Management, 5IconSWM 2015. Procedia Environmental Sciences 35 201 – 208, 2016.
55. Decharat, S. Mercury Exposure among Garbage Workers in Southern Thailand. *Safety and health at work* **2012**, 3(4), 268–277.
56. De Felice, B, Nappi, C, Zizolfi, B, Guida, M, Di Spiezio Sardo, A, Bifulco, G, Guida, M. Telomere shortening in women resident close to waste landfill sites. *Gene* **2012**, 500(1), 101–106.
57. Demesouka, OE, Anagnostopoulos, KP, Sisko, E. Spatial multicriteria decision support for robust land–use suitability: The case of landfill site selection in Northeastern Greece. *European Journal of Operational Research* **2019**, 272, 574–586.
58. Di Ciaula, A. Emergency visits and hospital admissions in aged people living close to a gas–fired power plant. *European Journal of Internal Medicine* **2012**, 23, e53–e58.
59. Diener, S, Zurbrügg, C, Tockner, K. Bioaccumulation of heavy metals in the black soldier fly, Hermetia illucens and effects on its life cycle. *Journal of Insects as Food and Feed* **2015**, 1 (4), 261–270.
60. Domingo, JL, Nadal, M. Domestic waste composting facilities: A review of human health risks. *Environment International* **2009**, 35, 382–389.
61. Dou, Z, Toth, JD, Westendorf, ML. Food waste for livestock feeding: Feasibility, safety, and sustainability implications. *Global Food Security* **2018**, 17, 154–161.
62. Douglas, P, Bakolis, I, Fecht, D, Pearson, C, Leal Sanchez, M, Kinnersley, R, de Hoogh, K, Hansell, AL. Respiratory hospital admission risk near large composting facilities. *Int J Hyg Environ Health* **2016**, 219 (4–5), 372–9.

63. Dounias, G, Kypraiou, E, Rachiotis, G, Tsovili, E, Kostopoulos, S. Prevalence of hepatitis B virus markers in municipal solid waste workers in Keratsini (Greece). *Occupational medicine* **2005**, 55 (1), 60–63.
64. Dubuis, ME, M'Bareche, H, Veillette, M, Bakhiyi, B, Zayed, J, Lavoie, J, Duchaine, C. Bioaerosols concentrations in working areas in biomethanization facilities. *Journal of the Air & Waste Management Association* **2017**, 67 (11), 1258–1271.
65. Durmusoglu, E, Taspinar, F, Karademir, A. Health risk assessment of BTEX emissions in the landfill environment. *Journal of Hazardous Materials* **2010**, 176, 870–877.
66. Elliott, P, Richardson, S, Abellan, JJ, Thomson, A, de Hoogh, C, Jarup, L, Briggs, DJ. Geographic density of landfill sites and risk of congenital anomalies in England. *Occup Environ Med* **2009**, 66, 81–89.
67. Engkvist, I. Working conditions at recycling centres in Sweden – Physical and psychosocial work environment. *Applied Ergonomics* **2010**, 41, 347–354.
68. Essien, JP, Inam, ED, Ikpe, DI, Udofia, GE, Benson, NU. Ecotoxicological status and risk assessment of heavy metals in municipal solid wastes dumpsite impacted soil in Nigeria. *Environmental Nanotechnology, Monitoring & Management* **2019**, 11, 100215.
69. Fazzo, L, Minichilli, F, Santoro, M, Ceccarini, A, Della Seta, M, Bianchi, F, Comba, P, Martuzzi, M. Hazardous waste and health impact: a systematic review of the scientific literature. *Environ Health* **2017**, 16 (1), 107.
70. Federico, M, Pirani, M, Rashid, I, Caranci, N, Cirilli, C. Cancer incidence in people with residential exposure to a municipal waste incinerator: an ecological study in Modena (Italy), 1991–2005. *Waste Management* **2010**, 30 (7), 1362–1370.
71. Ferronato, N, Torretta, V. Waste Mismanagement in Developing Countries: A Review of Global Issues. *Int. J. Environ. Res. Public Health* **2019**, 16, 1060.
72. Font X, Artola A, Sánchez A. Detection, composition and treatment of volatile organic compounds from waste treatment plants. *Sensors* **2011**, 11 (4), 4043–4059.
73. Forastiere, F, Badaloni, C, de Hoogh, K, von Kraus, MK, Martuzzi, M, Mitis, F, Palkovicova, L, Porta, D, Preiss, P., Ranzi, A, Perucci, CA, Briggs, D. Health impact assessment of waste management facilities in three European countries. *Environmental health* **2011**, 10, 53.
74. Forti, JC, Lima, PG, Reis, AR, dos Santos, FA, Braga Junior, SS. Analysis of heavy metals and aromatics compounds in soil layers of a sanitary landfill. *Environmental Quality Management* **2019**, 28, 123–130.
75. Galès, A, Bru–Adan, V, Godon, J, Delabre, K, Catala, P, Ponthieux, A, Chevallier, M, Birot, E, Steyer, J, Wéry, N. Predominance of single bacterial cells in composting bioaerosols. *Atmos. Environ.* **2015**, 107, 225–232.
76. Garcés–Ordóñez, O, Castillo–Olaya, VA, Granados–Briceño, AF, Blandón García, LM, Espinosa Díaza, LF. Marine litter and microplastic pollution on mangrove soils of the

Ciénaga Grande de Santa Marta, Colombian Caribbean. *Marine Pollution Bulletin* **2019**, 145, 455–462.
77. García–Pérez, J, Fernández–Navarro, P, Castelló, A, López–Cima, MF, Ramis, R, Boldo, E, López–Abente, G. Cancer mortality in towns in the vicinity of incinerators and installations for the recovery or disposal of hazardous waste. *Environment international* **2013**, 51, 31–44.
78. Gatti, MG, Bechtold, P, Campo, L, Barbieri, G, Quattrini, G, Ranzi, A, Sucato, S, Olgiati, L, Polledri, E, Romolo, M, Iacuzio, L, Carrozzi, G, Lauriola, P, Goldoni, CA, Fustinoni, S. Human biomonitoring of polycyclic aromatic hydrocarbonsand metals in the general population residing near the municipal solid waste incinerator of Modena, Italy. *Chemosphere* **2017**, 186, 546–557.
79. Ghosh, RE, Freni–Sterrantino, A, Douglas, P, Parkes, B, Fecht, D, de Hoogh, K, Fuller, G, Gulliver, J, Font, A, Smith, RB, Blangiardo, M, Elliott, P, Toledano, MB, Hansell, AL. Fetal growth, stillbirth, infant mortality and other birth outcomes near UK municipal waste incinerators; retrospective population based cohort and case–control study. *Environment International* **2019**, 122, 151–158.
80. Gilbreath, S, Kass, PH. Adverse birth outcomes associated with open dumpsites in Alaska Native Villages. *Am J Epidemiol* **2006**, 164 (6), 518–528.
81. Gilbreath, S, Kass, PH. Fetal and neonatal deaths and congenital anomalies associated with open dumpsites in Alaska Native villages. *Int J Circumpolar Health* **2006**, 65 (2), 133–147.
82. Giusti, L. A review of waste management practices and their impact on human health. *Waste Management* **2009**, 29, 2227–2239.
83. Gouveia, N, do Prado, RR. Health risks in areas close to urban solid waste landfill sites. *Rev Saude Publica* **2010**, 44 (5), 859–866.
84. Govasmark, E, Stäb, J, Holen, B, Hoornstra, D, Nesbakk, T, Salkinoja–Salonen, M. Chemical and microbiological hazards associated with recycling of anaerobic digested residue intended for agricultural use. *Waste Management* **2011**, 31(12), 2577–2583.
85. Guleira, A, Chakma, S. Probabilistic human health risk assessment of groundwater contamination due to metal leaching: A case study of Indian dumping sites. *Human and Ecological Risk Assessment: An International Journal* **2019**.
86. Gumede, PR, Savage, MJ. Respiratory health effects associated with indoor particulate matter (PM2.5) in children residing near a landfill site in Durban, South Africa. *Air Qual Atmos Health* **2017**, 10, 853–860.
87. Gutberlet, J, Baeder, AM, Pontuschka, NN, Felipone, SMN, Dos Santos, TLF. Participatory Research Revealing the Work and Occupational Health Hazards of Cooperative Recyclers in Brazil. *Int. J. Environ. Res. Public Health* **2013**, 10, 4607–4627.

88. Gwisai, RD, Areola, O, Segosebe, E. Respiratory and occupational health problems of scavengers and landfill employees in a municipal landfill site in Lobatse, Botswana. *Journal of Sustainable Development in Africa* **2014**, 16 (1).
89. Haddad, A, Moqbel, S. Modeling of dioxin levels in pine needles exposed to solid waste open combustion emissions. Waste Management **2018**, 79, 510–515.
90. Hafeez, S, Mahmood, A, Syed, JH, Li, J, Ali, U, Malik, RN, Zhang, G. Waste dumping sites as a potential source of POPs and associated health risks in perspective of current waste management practices in Lahore city, Pakistan. *Science of the total environment* **2016**, 562, 953–961.
91. Hargreaves, JC, Adl, MS, Warman, PR. A review of the use of composted municipal solid waste in agriculture. *Agriculture, Ecosystems and Environment* **2008**, 123, 1–14.
92. Harpham, T. Urban health in developing countries: What do we know and where do we go? *Health & Place* **2009**, 15, 107–116.
93. He, Z, Li, G, Chen, J, Huang, Y, An, T, Zhang, C. Pollution characteristics and health risk assessment of volatile organic compounds emitted from different plastic solid waste recycling workshops. *Environment international* **2015**, 77, 85–94.
94. Heidari, R, Yazdanparast, R, Jabbarzadeh, A. Sustainable design of a municipal solid waste management system considering waste separators: A real–world application. *Sustainable Cities and Society* **2019**, 47, 101457.
95. Heldal, KK, Madsø, L, Eduard, W. Airway inflammation among compost workers exposed to actinomycetes spores. *Annals of agricultural and environmental medicine* **2015**, 22 (2), 253–258.
96. Heaney, CD, Wing, S, Campbell, RL, Caldwell, D, Hopkins, B, Richardson, D, Yeatts, K, 2011. Relation between malodor, ambient hydrogen sulfide, and health in a community bordering a landfill. *Environ Res* **2011**, 111 (6), 847–852.
97. Hellström, A, Nilsson, ML, Kylin, H. Current–use and organochlorine pesticides and polychlorinated biphenyls in the biodegradable fraction of source separated household waste, compost, and anaerobic digest. *Bulletin of environmental contamination and toxicology* **2011**, 86 (1), 60–64.
98. Hoffmeyer, F, van Kampen, V, Taeger, D, Deckert, A, Rosenkranz, N, Kaßen, M, Schantora, AL, Brüning, T, Raulf, M, Bünger, J. Prevalence of and relationship between rhinoconjunctivitis and lower airway diseases in compost workers with current or former exposure to organic dust. *Annals of agricultural and environmental medicine* **2014**, 21(4), 705–711.
99. Ihedioha, JN, Ukoha, PO, Ekere, NR. Ecological and human health risk assessment of heavy metal contamination in soil of a municipal solid waste dump in Uyo, Nigeria. *Environmental geochemistry and health* **2017**, 39 (3), 497–515.

100. Inyang, MP. Health and safety risks amongst the municipal solid waste collectors in port harcourt metropolis of the Niger Delta region of Nigeria. Proceeding of the International Conference "Waste Management, Environmental Geotechnology and Global Sustainable Development, Ljubljana, SLOVENIA, August 28. – 30., 2007.
101. Ionescu, G, Zardi, D, Tirler, W, Rada, EC, Ragazzi, M. A critical analysis of emissions and atmospheric dispersion of pollutants from plants for the treatment of residual municipal solid waste. *U.P.B. Sci. Bull.* **2012**, 74 (4).
102. Jarup, L, Morris, S, Richardson, S, Briggs, D, Cobley, N, de Hoogh, C, Gorog, K, Elliott, P. Down syndrome in births near landfill sites. *Prenat Diagn* **2007**, 27 (13), 1191–1196.
103. Jayakrishnan, T, Jeeja, MC, Bhaskar, R. Occupational health problems of municipal solid waste management workers in India. *International Journal of Environmental Health Engineering* **2013**, 2 (3).
104. Jeong, BY, Lee, S, Lee, JD. Workplace Accidents and Work–related Illnesses of Household Waste Collectors. *Saf Health Work* **2016**, 7 (2), 138–42.
105. Kazaure, MB. Survey on SWM for Sustainable Development and Public Health in Dutse Metropolis, Jigawa State, Nigeria. Proceedings of the International Conference on Solid Waste Management, 5IconSWM 2015. Procedia Environmental Sciences 2016, 35, 57–64.
106. Khalil, C, Al Hageh, C, Korfali, S, Khnayzer, R. S. Municipal leachates health risks: Chemical and cytotoxicity assessment from regulated and unregulated municipal dumpsites in Lebanon. *Chemosphere* **2018**, 208, 1–13.
107. Kloppenborg, SCh,, Brandt, UK, Gulis, G, Ejstrud, B. Risk of congenital anomalies in the vicinity of waste landfills in Denmark; an epidemiological study using GIS. *Cent Eur J Public Health* **2005**, 13(3), 137–143.
108. Kret, J, Dalidowitz Dame, L, Tutlam, N, DeClue, RW, Schmidt, S, Donaldson, K, Lewis, R, Rigdon, SE, Davis, S, Zelicoff, A, King, C, Wang, Y, Patrick, S, Khan, F. A respiratory health survey of a subsurface smoldering landfill. *Environmental research* **2018**, 166, 427–436.
109. Krystosik, A., Njoroge, G., Odhiambo, L., Forsyth, J.E., Mutuku, F., LaBeaud, A.D. Solid Wastes Provide Breeding Sites, Burrows, and Food for Biological Disease Vectors, and Urban Zoonotic Reservoirs: A Call to Action for Solutions–Based Research. *Frontiers in public health* **2020**, 7, 405.
110. Kumari, K, Kumar, S, Rajagopal, V, Khare, A, Kumar, R. Emission from open burning of municipal solid waste in India. *Environ Technol.* **2019**, 40 (17), 2201–2214.
111. Lal, RM, Nagpure, AS, Luo, L, Tripathi, SN, Ramaswami, A, Bergin, MH, Russell, AG. Municipal solid waste and dung cake burning: discoloring the Taj Mahal and human health impacts in Agra. *Environ. Res. Lett.* **2016**, 11.

112. Leal Filho, W, Havea, PH, Balogun, AL, Boenecke, J, Maharaj, AA, Ha'apio, M, Hemstock, SL. Plastic debris on Pacific Islands: ecological and health implications. *Sci. Total Environ.* **2019**, 670, 181–187.
113. Leal Filho, W, Saari U, Fedoruk, M, Iital, A, Moora, H, Kloga, M, Voronova, V. An overview of the problems posed by plastic products and the role of extended producer responsibility in Europe. *Journal of Cleaner Production* **2019**, 214, 550–558.
114. Lenis BallesterosI, V, López Arango, YL, Cuadros Urrego, YM. Health and informal work conditions among recyclers in the rural area of Medellin, Colombia, 2008. *Revista de saude publica* **2012**, 46 (5), 866–874.
115. Li, J, Dong, H, Sun, J, Nie, J, Zhang, S, Tang, J, Chen, Z. Composition profiles and health risk of PCDD/F in outdoor air and fly ash from municipal solid waste incineration and adjacent villages in East China. *Science of the total environment* **2016**, 571, 876–882.
116. Li, J, Zhang, Y, Sun, T, Hao, H, Wu, H, Wang, L, Chen, Y, Xing, L, Niu, Z. The health risk levels of different age groups of residents living in the vicinity of municipal solid waste incinerator posed by PCDD/Fs in atmosphere and soil. *Science of the total environment* **2018**, 631–632, 81–91.
117. Lilford, RJ, Oyebode, O, Satterthwaite, D, Melendez–Torres, GJ, Chen, YF, Mberu, B, Watson, SI, Sartori, J, Ndugwa, R, Caiaffa, W, Haregu, T, Capon, A, Saith, R, Ezeh, A. Improving the health and welfare of people who live in slums. *The Lancet* **2017**, 389 (10068), 559–570.
118. Lin, CM, Li, CY, Mao, IF. Birth outcomes of infants born in areas with elevated ambient exposure to incinerator generated PCDD/Fs. *Environment international* **2006**, 32 (5), 624–629.
119. Liu, LJ, Chen, XC, Fu, JP, Qing, X, Huang, JQ, Han, JL. Male workers' exposure characteristics of ΣPCDD/F from a municipal solid waste incinerator in south China through hair analysis. *Ecotoxicology and Environmental Safety* **2019**, 178, 105–112.
120. Liu, Y, Kong, F, Santibanez Gonzalez, EDR. Dumping, waste management and ecological security: Evidence from England. *Journal of Cleaner Production* **2017**, 167, 1425–1437.
121. Liu, J, Pankhurst, LJ, Deacon, LJ, Abate, W, Hayes, ET, Drew, GH, Longhurst, PJ., Pollard, S, Longhurst, J, Tyrrel, SF, Jackson, SK. Evaluation of inflammatory effects of airborne endotoxin emitted from composting sources. *Environmental toxicology and chemistry* **2011**, 30(3), 602–606.
122. Liu, Y, Liu, Y, Li, H, Fu, X, Guo, H, Meng, R, Lu, W, Zhao, M, Wang, H. Health risk impacts analysis of fugitive aromatic compounds emissions from the working face of a municipal solid waste landfill in China. *Environment international* **2016**, 97, 15–27.

123. Lonati, G, Cernuschi, S, Giugliano, M, Grosso, M. Health risk analysis of PCDD/F emissions from MSW incineration: comparison of probabilistic and deterministic approaches. *Chemosphere* **2007**, 67 (9), S334–343.
124. Ma, J, Kouznetsova, M, Lessner, L, Carpenter, DO. Asthma and infectious respiratory disease in children—correlation to residence near hazardous waste sites. *Paediatric respiratory reviews* **2007**, 8 (4), 292–298.
125. Ma, W, Tai, L, Qiao, Z, Zhong, L, Wang, Z, Fu, K, Chen, G. Contamination source apportionment and health risk assessment of heavy metals in soil around municipal solid waste incinerator: A case study in North China. *Science of the total environment* **2018**, 631–632, 348–357.
126. Marfe, G, Di Stefano, C. The evidence of toxic wastes dumping in Campania, Italy. *Crit Rev Oncol Hematol.* **2016**, 105, 84–91.
127. Mari, M, Ferré–Huguet, N, Nadal, M, Schuhmacher, M, Domingo, JL. Temporal Trends in Metal Concentrations in Soils and Herbage Collected Near a Municipal Waste Incinerator: Human Health Risks. *Human and Ecological Risk Assessment* **2007**, 13, 457–472.
128. Markic, A, Niemand, C, Bridson, JH, Mazouni–Gaertner, N, Gaertner, JC, Eriksen, M, Bowen, M. Double trouble in the South Pacific subtropical gyre: Increased plastic ingestion by fish in the oceanic accumulation zone. *Marine pollution bulletin* **2018**, 136, 547–564.
129. Martuzzi, M, Mitis, F, Bianchi, F, Minichilli, F, Comba, P, Fazzo, L. Cancer mortality and congenital anomalies in a region of Italy with intense environmental pressure due to waste. *Occupational and environmental medicine* **2009**, 66 (11), 725–732.
130. Massos, A, Turner, A. Cadmium, lead and bromine in beached microplastics. *Environ Pollut* **2017**, 227, 139–145.
131. Mataloni, F., Badaloni, C., Golini, M.N., Bolignano, A., Bucci, S., Sozzi, R., Forastiere, F., Davoli, M., Ancona C. Morbidity and mortality of people who live close to municipal waste landfills: a multisite cohort study. *International Journal of Epidemiology* **2016**, 45 (3), 806–815.
132. Mattiello, A, Chiodini, P, Bianco, E, Forgione, N, Flammia, I, Gallo, C, Pizzuti, R, Panico, S. Health effects associated with the disposal of solid waste in landfills and incinerators in populations living in surrounding areas: a systematic review. *International Journal of Public Health* **2013**, 58 (5), 725–35.
133. Mbareche, H, Veillette, M, Dubuis, MÈ, Bakhiyi, B, Marchand, G, Zayed, J, Lavoie, J, Bilodeau, GJ, Duchaine, C. Fungal bioaerosols in biomethanization facilities. *Journal of the Air & Waste Management Association* **2018**, 68 (11), 1198–1210.

134. McGoran, AR, Cowie, PR, Clark, PF, McEvoy, JP, Morritt, D. Ingestion of plastic by fish: A comparison of Thames Estuary and Firth of Clyde populations. *Marine pollution bulletin* **2018**, 137, 12–23.
135. Mehrdad, R, Majlessi–Nasr, M, Aminian, O, Sharifian, SA, Malekahmadi, F. Musculoskeletal disorders among municipal solid waste workers. *Acta Medica Iranica* **2008**, 46 (3).
136. Miranda, DA, de Carvalho–Souza, GF. Are we eating plastic–ingesting fish?. *Marine pollution bulletin* **2016**, 103 (1–2), 109–114.
137. Mishra, S, Tiwary, D, Ohri, A, Agnihotri, AK. Impact of Municipal Solid Waste Landfill leachate on groundwater quality in Varanasi, India. *Groundwater for Sustainable Development* **2019**, 9, 100230.
138. Moletta M, Delgenes JP, Godon JJ. Differences in the aerosolization behavior of microorganisms as revealed through their transport by biogas. *Sci Total Environ.* **2007**, 379 (1), 75–88.
139. Morsi, RZ, Safa, R, Baroud, SF, Fawaz, CN, Farha, JI, El–Jardali, F, Chaaya, M. The protracted waste crisis and physical health of workers in Beirut: a comparative cross–sectional study. *Environ Health* **2017**, 16(1), 39.
140. Moy, P, Krishnan, N, Ulloa, P, Cohen, S, Brandt–Rauf, PW. Options for management of municipal solid waste in New York City: a preliminary comparison of health risks and policy implications. *Journal of Environmental Management* **2008**, 87 (1), 73–79.
141. Murphy, S, Gaffney, MT, Fanning, S, Burgess, C. M. Potential for transfer of Escherichia coli O157:H7, Listeria monocytogenes and Salmonella Senftenberg from contaminated food waste derived compost and anaerobic digestate liquid to lettuce plants. *Food microbiology* **2016**, 59, 7–13.
142. Mustafa, MF, Liu, Y, Duan, Z, Guo, H, Xu, S, Wang, H, Lu, W. Volatile compounds emission and health risk assessment during composting of organic fraction of municipal solid waste. *Journal of Hazardous Materials* **2017**, 327, 35–43.
143. Nadal, M, Mari, M, Schuhmacher, M, Domingo, JL. Monitoring dioxins and furans in plasma of individuals living near a hazardous waste incinerator: Temporal trend after 20 years. *Environmental Research* **2019**, 173, 207–211.
144. Nagpure, AS, Ramaswami, A, Russell, A. Characterizing the Spatial and Temporal Patterns of Open Burning of Municipal Solid Waste (MSW) in Indian Cities. *Environmental science & technology* **2015**, 49(21), 12904–12912.
145. Naja, GM, Alary, R, Bajeat, P, Bellenfant, G, Godon, J, Jaeg, J, Keck, G, Lattes, A, Leroux, C, Modelon, H, Moletta–Denat, M, Ramalho, O, Rousselle, C, Wenisch, S, Zdanevitch, I. Assessment of biogas potential hazards. *Renewable Energy* **2011**, 36 (12), 3445–3451.

146. Nartey, VK, Hayford, EK, Ametsi, SK. Assessment of the Impact of Solid Waste Dumpsites on Some Surface Water Systems in the Accra Metropolitan Area, Ghana. *Journal of Water Resource and Protection* **2012**, 4, 605–615.
147. Ncube, F, Ncube, EJ, Voyi, K. A systematic critical review of epidemiological studies on public health concerns of municipal solid waste handling. *Perspectives in Public Health* **2017**, 137 (2), 102–108.
148. Neuhaus, J, Shehata, AA, Krüger, M. Detection of pathogenic clostridia in biogas plant wastes. *Folia microbiologica* **2015**, 60 (1), 15–19.
149. Nguendo Yongsi, HB, Herrmann, MT, Lutumba Ntetu, A, Sietchiping, R, Bryant, C. Environmental Sanitation and Health Risks in Tropical Urban Settings: Case Study of Household Refuse and Diarrhea in Yaoundé–Cameroon. *Int. J. Soci. Sci* **2008**, 3, 220–229.
150. Nie, E, Zheng, G, Shao, Z, Yang, J, Chen, T. Emission characteristics and health risk assessment of volatile organic compounds produced during municipal solid waste composting. *Waste Management* **2018**, 79, 188–195.
151. Nikaeen, M, Hatamzadeh, M, Hasanzadeh, A, Sahami, E, Joodan, I. Bioaerosol emissions arising during application of municipal solid–waste compost. *Aerobiologia* **2009**, 25, 1–6.
152. Odewabi, AO, Ogundahunsi, OA, Ekor, M. Effect of exposure to solid wastes in relation to employment duration on some important markers of health and disease in waste management workers of Ogun State in southwest Nigeria. *Human & experimental toxicology* **2013**, 32 (12), 1231–1244.
153. Ogunbanjo, O, Onawumi, O, Gbadamosi, M, Ogunlana, A, Anselm, O. Chemical speciation of some heavy metals and human health risk assessment in soil around two municipal dumpsites in Sagamu, Ogun state, Nigeria. Chemical Speciation & Bioavailability **2016**, 28 (1–4), 142–151.
154. Oguntoke, O, Emoruwa, FO, Taiwo, MA. Assessment of air pollution and health hazard associated with sawmill and municipal waste burning in Abeokuta Metropolis, Nigeria. *Environmental science and pollution research international* **2019**, 26 (32), 32708–32722.
155. Ogunrinola, IO, Adepegba, EO. Health and Economic Implications of Waste Dumpsites in Cities: The Case of Lagos, Nigeria. *International Journal of Economics and Finance* **2012**, 4, 4.
156. Okedere, OB, Olalekan, AP, Fakinle, BS, Elehinafe, FB, Odunlami, OA, Sonibare, JA. Urban air pollution from the open burning of municipal solid waste. *Environ Qual Manage* **2019**, 28, 67–74.
157. Orioli, IM, Mastroiacovo, P, López–Camelo, JS, Saldarriaga, W, Isaza, C, Aiello, H, Zarante I, Castilla, EE. Clusters of sirenomelia in South America. *Birth Defects Research (Part A)* **2009**, 85, 112–118.

158. Owamah, HI, Dahunsi, SO, Oranusi, US, Alfa, M I. Fertilizer and sanitary quality of digestate biofertilizer from the co–digestion of food waste and human excreta. *Waste Management* **2014**, 34(4), 747–752.
159. Owusu–Sekyere, E. Scavenging for wealth or death? Exploring the health risk associated with waste scavenging in Kumasi, Ghana. *Ghana Journal of Geography* **2014**, 6, 63–80.
160. Pahari, AK, Dasgupta, D, Patil, RS, Mukherji, S. Emission of bacterial bioaerosols from a composting facility in Maharashtra, India. *Waste Management* **2016**, 53, 22–31.
161. Paladino, O, Massabò, M. Health risk assessment as an approach to manage an old landfill and to propose integrated solid waste treatment: A case study in Italy. *Waste management* **2017**, 68, 344–354.
162. Palmer, SR, Dunstan, FD, Fielder, H, Fone, DL, Higgs, G, Senior, ML. Risk of congenital anomalies after the opening of landfill sites. *Environ Health Perspect* **2005**, 113 (10), 1362–1365.
163. Parera, J, Serra–Prat, M, Palomera, E, Mattioli, L, Abalos, M, Rivera, J, Abad, E. Biological monitoring of PCDD/Fs and PCBs in the City of Mataró. A population–based cohort study (1995–2012). *Science of the Total Environment* **2013**, 461–462, 612–617.
164. Parkes, B, Hansell, AL, Ghosh, RE, Douglas, P, Fecht, D, Wellesley, D, Kurinczuk, JJ, ì Rankin, J, de Hoogh, K,. Fuller, GW, Elliott, P, Toledano, MB. Risk of congenital anomalies near municipal waste incinerators in England and Scotland: Retrospective population–based cohort study. *Environment International* **2020**, 134, 104845.
165. Pearson, C, Littlewood, E, Douglas, P, Robertson, S, Gant, TW, Hansell, AL. Exposures and health outcomes in relation to bioaerosol emissions from composting facilities: a systematic review of occupational and community studies. *Journal of toxicology and environmental health. Part B* **2015**, 18 (1), 43–69.
166. Perez, HR, Frank, AL, Zimmerman, NJ. Health Effects Associated With Organic Dust Exposure During the Handling of Municipal Solid Waste. *Indoor and Built Environment* **2006**, 15, 207–212.
167. Peter, AE, Nagendra, SMS, Nambi, IM. Comprehensive analysis of inhalable toxic particulate emissions from an old municipal solid waste dumpsite and neighborhood health risks. *Atmos Pollut Res* **2018**, 9, 1021–1031.
168. Petrovic, M, Sremacki, M, Radonic, J, Mihajlovic, I, Obrovski, B, Vojinovic Miloradov, M. Health risk assessment of PAHs, PCBs and OCPs in atmospheric air of municipal solid waste landfill in Novi Sad, Serbia. *The Science of the total environment* **2018**, 644, 1201–1206.
169. Pollard, SJ, Smith, R, Longhurst, PJ, Eduljee, GH, Hall, D. Recent developments in the application of risk analysis to waste technologies. *Environment international* **2006**, 32 (8), 1010–1020.

170. Poole, CJM, Basu, S. Systematic Review: Occupational illness in the waste and recycling sector. *Occup Med* **2017**, 67 (8), 626–636.
171. Porta, D, Milani, S, Lazzarino, AI, Perucci, CA, Forastiere, F. Systematic review of epidemiological studies on health effects associated with management of solid waste. *Environmental Health* **2009**, 8, 60.
172. Prasad Raju, H, Partheeban, P. Mobile monitoring of air pollution emanating from burning of MSW: impact on human health – A case study. *International journal of earth sciences and engineering* **2014**, 7 (5), 1799–1805.
173. Pujara, Y, Pathak, P, Sharma, A, Govani, J. Review on Indian Municipal Solid Waste Management practices for reduction of environmental impacts to achieve sustainable development goals. *Journal of environmental management* **2019**, 248, 109238.
174. Purschke, B, Scheibelberger, R, Axmann, S, Adler, A, Jäger, H. Impact of substrate contamination with mycotoxins, heavy metals and pesticides on the growth performance and composition of black soldier fly larvae (Hermetia illucens) for use in the feed and food value chain. *Food additives & contaminants. Part A* **2017**, 34 (8), 1410–1420.
175. Rachiotis, G, Papagiannis, D, Thanasias, E, Dounias, G, Hadjichristodoulou, C. Hepatitis A Virus Infection and the Waste Handling Industry: A Seroprevalence Study. *Int. J. Environ. Res. Public Health* **2012**, 9, 4498–4503.
176. Rada, EC, Ferrari, A, Ragazzi, M, Schiavon, M, Torretta, V. PCDD/Fs environmental impact from an anaerobic digestion treatment. *Progress in Industrial Ecology* **2016**, 10 (4), 370–381.
177. Rada, EC, Franzinelli, A, Ragazzi, M, Panaitescu, V, Apostol, T. Modelling of PCDD/F release from MSW bio–drying. *Chemosphere* **2007**, 68, 1669–1674.
178. Rada, EC, Ragazzi, M. Critical analysis of PCDD/F emissions from anaerobic digestion. *Water Science & Technology* **2008**, 58 (9), 1721–1725.
179. Ranzi, A, Fano, V, Erspamer, L, Lauriola, P, Perucci, CA, Forastiere, F. (2011). Mortality and morbidity among people living close to incinerators: a cohort study based on dispersion modeling for exposure assessment. *Environmental health* **2011**, 10, 22.
180. Ravindra, K, Kaur, K, Mor, S. Occupational exposure to the municipal solid waste workers in Chandigarh, India. *Waste management & research* **2016**, 34 (11), 1192–1195.
181. Reddy, EM, Yasobant, S. Musculoskeletal disorders among municipal solid waste workers in India: A cross–sectional risk assessment. *Journal of family medicine and primary care* **2015**, 4 (4), 519–524.
182. Reyna–Bensusan, N, Wilson, DC, Smith, SR. Uncontrolled burning of solid waste by households in Mexico is a significant contributor to climate change in the country. *Environ Res* **2018**, 163, 280–288.

183. Rios, LM, Moore, C, Jones, PR. Persistent organic pollutants carried by synthetic polymers in the ocean environment. *Marine pollution bulletin* **2007**, 54 (8), 1230–1237.
184. Roberts, RJ, Chen, M. Waste incineration—how big is the health risk? A quantitative method to allow comparison with other health risks. *Journal of public health* **2006**, 28 (3), 261–266.
185. Robertson, S, Douglas, P, Jarvis, D, Marczylo, E. Bioaerosol exposure from composting facilities and health outcomes in workers and in the community: A systematic review update. *International Journal of Hygiene and Environmental Health* **2019**, 222 (3), 364–386.
186. Rounsefell, BD, O'Sullivan, CA, Chinivasagam, N, Batstone, D, Clarke, WP. Fate of pathogen indicators in a domestic blend of food waste and wastewater through a two–stage anaerobic digestion system. *Water science and technology* **2013**, 67 (2), 366–373.
187. Rovira, J, Nadal, M, Schuhmacher, M, Domingo, JL. Concentrations of trace elements and PCDD/Fs around a municipal solid waste incinerator in Girona (Catalonia, Spain). Human health risks for the population living in the neighborhood. *Science of the total environment* **2018**, 630, 34–45.
188. Rovira, J, Vilavert, L, Nadal, M, Schuhmacher, M, Domingo, JL. Temporal trends in the levels of metals, PCDD/Fs and PCBs in the vicinity of a municipal solid waste incinerator. Preliminary assessment of human health risks. *Waste Management* **2015**, 43, 168–175.
189. Ruggieri, F, Alimonti, A, Bena, A, Pino, A, Orengia, M, Farina, E, Salamina, G, Procopio, E, Gandini, M, Cadum, E, Bocca, B. Human biomonitoring health surveillance for metals near a waste–to–energy incinerator: The 1–year post–operam study. *Chemosphere* **2019**, 225, 839–848.
190. Russi, MB, Borak, JB, Cullen, MR. An examination of cancer epidemiology studies among populations living close to toxic waste sites. *Environmental health* **2008**, 7, 32.
191. Ryckebosch, E, Drouillon, M, Vervaeren, H. Techniques for transformation of biogas to biomethane. *Biomass and Bioenergy* **2011**, 35, 1633–1645.
192. Salemdeeb, R, Zu Ermgassen, EK, Kim, MH, Balmford, A, Al–Tabbaa, A. Environmental and health impacts of using food waste as animal feed: a comparative analysis of food waste management options. *Journal of cleaner production* **2017**, 140, 871–880.
193. Sankoh, FP, Yan, X, Tran, Q. Environmental and Health Impact of Solid Waste Disposal in Developing Cities: A Case Study of Granville Brook Dumpsite, Freetown, Sierra Leone. *Journal of Environmental Protection* **2013**, 4, 665–670.
194. Silva, MM, Maldonado, GC, Castro, RO, de Sá Felizardo, J, Pereira Cardoso, R, Dos Anjos, RM, de Araújo, FV. Dispersal of potentially pathogenic bacteria by plastic debris in Guanabara Bay, RJ, Brazil. *Marine Pollution Bulletin* **2019**, 141, 561–568.
195. Shah, GM, Tufail, N, Bakhat, HF, Ahmad, I, Shahid, M, Hammad, HM, Nasim, W, Waqar, A, Rizwan, M, Dong, R. Composting of municipal solid waste by different

methods improved the growth of vegetables and reduced the health risks of cadmium and lead. *Environmental Science and Pollution Research* **2019**, 26 (6), 5463–5474.
196. Sharifi, Z, Hossaini, SMT, Renella, G. Risk assessment for sediment and stream water polluted by heavy metals released by a municipal solid waste composting plant. *Journal of Geochemical Exploration* **2016**, 169, 202–210.
197. Smith, SR, Lang, NL, Cheung, KH, Spanoudaki, K. Factors controlling pathogen destruction during anaerobic digestion of biowastes. *Waste management* **2005**, 25 (4), 417–425.
198. Song, Q, Li, J, Zeng, X. Minimizing the increasing solid waste through zero waste strategy. *Journal of Cleaner Production* **2015**, 104, 199–210.
199. Spinazzè, A, Borghi, F, Rovelli, S, Cavallo, DM. Exposure Assessment Methods in Studies on Waste Management and Health Effects: An Overview. *Environments* **2017**, 4, 19.
200. Sykes, P, Jones, K, Wildsmith, JD. Managing the potential public health risks from bioaerosol liberation at commercial composting sites in the UK: An analysis of the evidence base. *Resources, Conservation and Recycling* **2007**, 52, 410–424.
201. Suleman, Y, Darko ET, Agyemang–Duah, W. Solid Waste Disposal and Community Health Implications in Ghana: Evidence from Sawaba, Asokore Mampong Municipal Assembly. *Journal of Civil & Environmental Engineering* **2015**, 5 (6), 1000202.
202. Sultan, M, Waheed, S, Ali, U, Sweetman, AJ, Jones, KC, Malik, RN. Insight into occurrence, profile and spatial distribution of organochlorine pesticides in soils of solid waste dumping sites of Pakistan: Influence of soil properties and implications for environmental fate. *Ecotoxicology and environmental safety* **2019**, 170, 195–204.
203. Tait, PW, Brew, J, Che, A, Costanzo, A, Danyluk, A, Davis, M, Khalaf, A, McMahon, K, Watson, A, Rowcliff, K, Bowles, D. The health impacts of waste incineration: a systematic review. Australian and New Zealand journal of public health **2020**, 44 (1), 40–48.
204. Tambone, F, Scaglia, B, D'Imporzano, G, Schievano, A, Orzi, V, Salati, S, Adani, F. Assessing amendment and fertilizing properties of digestates from anaerobic digestion through a comparative study with digested sludge and compost. *Chemosphere* **2010**, 81 (5), 577–583.
205. Tao, Z, Dai, S, Chai, X. Mercury emission to the atmosphere from municipal solid waste landfills: A brief review. *Atmospheric Environment* **2017**, 170, 303–311.
206. Thakur, P, Ganguly, R, Dhulia, A. Occupational Health Hazard Exposure among municipal solid waste workers in Himachal Pradesh, India. *Waste Management* **2018**, 78, 483–489.
207. Thirarattanasunthon, P, Siriwong, W, Robson, M, Borjan, M. Health risk reduction behaviors model for scavengers exposed to solid waste in municipal dump sites in

Nakhon Ratchasima Province, Thailand. *Risk management and healthcare policy* **2012**, 5, 97–104.

208. Toufexi, E, Tsarpali, V, Efthimiou, I, Vidali, MS, Vlastos, D, Dailianis, S. Environmental and human risk assessment of landfill leachate: an integrated approach with the use of cytotoxic and genotoxic stress indices in mussel and human cells. *Journal of hazardous materials* **2013**, 260, 593–601.
209. Umlauf, G, Christoph, EH, Lanzini, L, Savolainen, R, Skejo, H, Bidoglio, G, Clemens, J, Goldbach, H, Scherer, H. PCDD/F and dioxin–like PCB profiles in soils amended with sewage sludge, compost, farmyard manure, and mineral fertilizer since 1962. *Environmental science and pollution research international* **2011**, 18 (3), 461–470.
210. Uriah, LA, Shehu, U. Environmental risk assessment of heavy metals content of municipal solid waste used as organic fertilizer in vegetable gardens on the Jos Plateau, Nigeria. *American Journal of Environmental Protection* **2014**, 3 (6–2), 1–13.
211. Vaccari, M, Vinti, G, Cesaro, A, Belgiorno, V, Salhofer, S, Dias, MI, Jandric, A. WEEE Treatment in Developing Countries: Environmental Pollution and Health Consequences—An Overview. Int. J. Environ. Res. Public Health 2019, 16, 1595.
212. Vaccari, M, Vinti, G, Tudor, T. An Analysis of the Risk Posed by Leachate from Dumpsites in Developing Countries. *Environments* **2018**, 5, 99.
213. Valerio, F. Environmental impacts of post–consumer material managements: Recycling, biological treatments, incineration. *Waste Management* **2010**, 30, 2354–2361.
214. Verma, R, Vinoda, KS, Papireddy, M, Gowda, ANS. Toxic Pollutants from Plastic Waste– A Review. Proceedings of the International Conference on Solid Waste Management, 5IconSWM 2015. Procedia Environmental Sciences 35, 701–708, 2016.
215. Viel, JF, Clément, MC, Hägi, M, Grandjean, S, Challier, B, Danzon, A. Dioxin emissions from a municipal solid waste incinerator and risk of invasive breast cancer: a population–based case–control study with GIS–derived exposure. *International Journal of Health Geographics* **2008**, 7, 4.
216. Viel, JF, Daniau, C, Goria, S, Fabre, P, de Crouy–Chanel, P, Sauleau, EA, Empereur–Bissonnet, P. Risk for non Hodgkin's lymphoma in the vicinity of French municipal solid waste incinerators. *Environmental health* **2008**, 7, 51.
217. Vilavert, L, Nadal, M, Schuhmacher, M, Domingo, JL. Long–term monitoring of dioxins and furans near a municipal solid waste incinerator: human health risks. *Waste Management & Research* **2012**, 30 (9), 908–916.
218. Vinceti, M, Malagoli, C, Fabbi, S, Teggi, S, Rodolfi, R, Garavelli, L, Astolfi, G, Rivieri, F. Risk of congenital anomalies around a municipal solid waste incinerator: a GIS–based case–control study. *Int J Health Geogr* **2009**, 8, 8.
219. Vinceti, M, Malagoli, C, Teggi, S, Fabbi, S, Goldoni, C, De Girolamo, G, Ferrari, P, Astolfi, G, Rivieri, F, Bergomi, M. Adverse pregnancy outcomes in a population exposed

to the emissions of a municipal waste incinerator. *Science of the total environment* **2008**, 407(1), 116–121.

220. Wang, Y, Li, J, An, D, Xi, B, Tang, J, Wang, Y, Yang, Y. Site selection for municipal solid waste landfill considering environmental health risks. *Resources, Conservation & Recycling* **2018**, 138 40–46.
221. World Health Organization (WHO), 2007. Population Health and Waste Management: Scientific Data and Policy Options. Report of a WHO Workshop, 29–30 March 2007, Rome, Italy.
222. World Health Organization (WHO), 2016. Waste and human health: evidence and needs. WHO Meeting Report: 5–6 November 2015. Bonn, Germany. Copenhagen: WHO Regional Office for Europe; 2016.
223. World Health Organization (WHO), 2019. Microplastics in drinking–water. Geneva: World Health Organization. ISBN 978–92–4–151619–8.
224. Xiong, X, Liu, X, Yu, I, Wang, L, Zhou, J, Sun, X, Rinklebe, J, Shaheen, SM, Ok, YS, Lin, Z, Tsang, D. Potentially toxic elements in solid waste streams: Fate and management approaches. *Environmental pollution* **2019**, 253, 680–707.
225. Xu, P, Chen, Z, Wu, L, Chen, Y, Xu, D, Shen, H, Han, J, Wang, X, Lou, X. Health risk of childhood exposure to PCDD/Fs emitted from a municipal waste incinerator in Zhejiang, China. *Science of the Total Environment* **2019**, 689, 937–944.
226. Xu, P, Wu, L, Chen, Y, Xu, D, Wang, X, Shen, H, Han, J, Fu, Q, Chen, Z, Lou, X. High intake of persistent organic pollutants generated by a municipal waste incinerator by breastfed infants. *Environmental Pollution* **2019**, 250, 662–668.
227. Yang, L, Liu, G, Zhu, Q, Zheng, M. Small–scale waste incinerators in rural China: Potential risks of dioxin and polychlorinated naphthalene emissions. *Emerging Contaminants* **2019**, 5, 31–34.
228. Yogesh, SD, Zodpey, SP. Respiratory morbidity among street sweepers working at Hanumannagar Zone of Nagpur Municipal Corporation, Maharashtra. *Indian journal of public health* **2008**, 52 (3), 147–149.
229. Yu, Y, Yu, Z, Sun, P, Lin, B, Li, L, Wang, Z, Ma, R, Xiang, M, Li, H, Guo, S. Effects of ambient air pollution from municipal solid waste landfill on children's non–specific immunity and respiratory health. *Environmental pollution* **2018**, 236, 382–390.
230. Zambon, P, Ricci, P, Bovo, E, Casula, A, Gattolin, M, Fiore, AR, Chiosi, F, Guzzinati, S. Sarcoma risk and dioxin emissions from incinerators and industrial plants: a population–based case–control study (Italy). *Environmental health* **2007**, 6, 19.
231. Zemba, SG, Binder, JJ, Ames, MR, Lester, RR. A Risk Assessment Framework for Evaluating Health Risks From New and Emerging Waste Management Technologies. Proceedings of the 18th Annual North American Waste–to–Energy Conference, Orlando, Florida, USA, May 11–13, 2010.

232. Zhang, G, Huang, X, Liao, W, Kang, S, Ren, M, Hai, J. Measurement of Dioxin Emissions from a Small–Scale Waste Incinerator in the Absence of Air Pollution Controls. *International journal of environmental research and public health* **2019**, 16 (7), 1267.
233. Zhang, H, Schuchardt, F, Li, G, Yang, J, Yang, Q. Emission of volatile sulfur compounds during composting of municipal solid waste (MSW). *Waste Management* **2013**, 33, 957–963.
234. Ziraba, AK, Haregu, TN, Mberu, B. A review and framework for understanding the potential impact of poor solid waste management on health in developing countries. *Archives of public health* **2016**, 74, 55.
235. Zolnikov, TR, da Silva, RC, Tuesta, AA, Marques, CP, Cruvinel, V. Ineffective waste site closures in Brazil: A systematic review on continuing health conditions and occupational hazards of waste collectors. *Waste Management* **2018**, 80, 26–39.
236. Zurbrügg, C, Caniato, M, Vaccari, M. How Assessment Methods Can Support Solid Waste Management in Developing Countries—A Critical Review. *Sustainability* **2014**, 6, 545–570.
